# A novel and facile synthesis of 3-(2-benzofuroyl)- and 3,6-bis(2-benzofuroyl)carbazole derivatives

**DOI:** 10.3762/bjoc.7.180

**Published:** 2011-11-17

**Authors:** Wentao Gao, Meiru Zheng, Yang Li

**Affiliations:** 1 Institute of Superfine Chemicals, Bohai University, Jinzhou 121000, China

**Keywords:** 2-benzofuroyl, carbazole, PEG-400, Rap–Stoermer reaction, salicylaldehydes, ultrasound-assisted

## Abstract

A facile synthesis of hitherto unreported 3-(2-benzofuroyl)carbazoles **3a**–**k**, 3,6-bis(2-benzofuroyl)carbazoles **5a**–**k**, and naphtho[2,1-*b*]furoylcarbazoles **3l** and **5l** is described. The synthesis mainly relies on the ultrasound-assisted Rap–Stoermer reaction of 3-chloroacetyl- (**1**) or 3,6-dichloroacetyl-9-ethyl-9*H*-carbazole (**4**) with various salicylaldehydes **2a**–**k** as well as 2-hydroxy-1-naphthaldehyde (**2l**) in CH_3_CN with the presence of PEG-400 as catalyst. The procedure offers easy access to benzofuroylcarbazoles in short reaction times and the products are obtained in moderate to good yields.

## Introduction

Carbazole, and especially heterocycle-containing carbazole derivatives, are embodied in many naturally occurring products [1–3] and display a broad spectrum of useful biological activities such as antitumor, antimitotic, and antioxidative activities [4–6]. They are also widely used as building blocks for new organic materials [7–10], and play a very important role in electroactive and photoactive devices [11–14]. Therefore, a number of methodologies for the construction of heterocycle-containing carbazoles have been reported in recent years [15–19]. Most heterocycle-containing carbazoles reported in the literature comprise a common heterocyclic ring moiety fused with a carbazole ring, such as pyridocarbazoles [20–21], thienocarbazoles [22–23], pyranocarbazoles, pyrrolocarbazoles [24–25], indolocarbazoles [26–28], and synthetic analogues thereof. However, there are very few reports in which the heterocyclic moiety is substituted with a carbazole unit. Hence the synthesis of such compounds is desirable [29–30].

On the other hand, the benzofuran derivatives are an important class of heterocyclic compounds that are known to possess important biological properties [31–33]. Especially, recent studies have shown that some benzofuroyl-based compounds display important biological properties as antimicrobial [34], anticonvulsant, anti-inflammatory [35], anti-tumor [36], and antifungal [37–38] activities. On account of these findings, extensive synthetic efforts have been devoted to the development of more novel and interesting benzofuroyl-based compounds [39–43].

We have recently reported the synthesis of quinolyl-substituted carbazoles [44] and benzofuranyl-substituted quinoline [45]. Thus, in light of the above findings and in the context of our ongoing work on the synthesis of new heterocyclic compounds, we found it an attractive idea to construct new prototypes combining both the carbazole ring system and benzofuran framework in the same molecule. Such compounds are not only synthetically challenging but may also be vitally important for pharmacological studies or in the realization of new medicinal properties. Therefore, we report herein the synthesis of a series of novel 3-(2-benzofuroyl)carbazoles and 3,6-bis(2-benzofuroyl)carbazoles.

## Results and Discussion

**Scheme 1:**
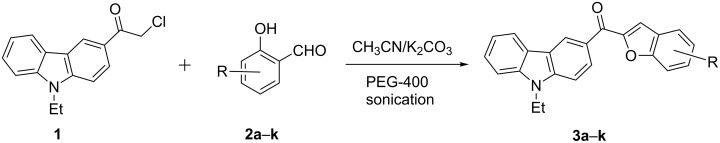
PEG-400 catalyzed ultrasound-assisted Rap–Stoermer synthesis of 3-(2-benzofuroyl)carbazoles **3a**–**k**.

In order to synthesize the targeted compounds through a facile and direct methodology, we devised a route that made use of the Rap–Stoermer reaction [46], and which could provide opportunity for the direct construction of 2-benzofuroyl-based compounds through base-mediated reaction of salicylaldehydes with α-haloketones. The synthetic route developed in our laboratory for the preparation of 3-(2-benzofuroyl)carbazoles **3a**–**k** by the Rap–Stoermer reaction of 3-chloroacetyl-9-ethyl-9*H-*carbazole (**1**) with a variety of salicylaldehydes **2a**–**k** is summarized in Scheme 1.

The Rap–Stoermer reaction was normally performed in alcoholic medium but often produced poor to moderate yields of benzofuran products [47–48]. Considering this fact, we conducted our own initial investigation towards the synthesis of **3a** according to reported methods under solvent-free [49] or solvent-free, microwave-irradiation conditions [50]. Unfortunately, it was found that the Rap–Stoermer reaction did not occur or gave intractable, complex mixtures (as observed by TLC), according to both methods. More recently, Shang et al. [51] described the base-mediated 4-dimethylaminopyridine (DMAP)-catalyzed Rap–Stoermer reaction for the synthesis of 2-benzofuroyl compounds in good yields between salicylaldehydes and halogenated ketones in water. Although the methodology is elegant and impressive, our attempts to follow the route to synthesize **3a** were also frustrated by the very complex mixture of the resulting products, from which we could not separate any desired products in appreciable yields. After many trials, we found that when the Rap–Stoermer reaction was carried out with PEG-400 (0.5 equiv) as catalyst in the presence of K_2_CO_3_ as base in refluxing CH_3_CN for 10 h, the desired benzofurans **3a** were obtained, but the attempt was still plagued by low yield. In this reaction the use of 0.5 equivalents of PEG-400 was found most suitable with **1** and **2a** to provide a maximum yield of **3a** of only 29%. There was no further improvement in the yields upon increasing the amount of catalyst or the reaction time. As a result, attempts to find an alternative approach are still very desirable.

Recently, the ultrasound technique has increasingly been used in synthetic organic chemistry. A large number of organic reactions can be carried out with a higher yield, in shorter reaction time and under milder conditions with the aid of ultrasonication. For example, Palimkar et al. [52] ever reported a facile ultrasound-promoted synthesis of benzo[*b*]furan derivatives. Accordingly, the versatility of the ultrasound technique prompted us to further experiment with this approach. Interestingly, we found that when the same reaction as above was adopted in conjunction with ultrasonic irradiation, an improvement in terms of yield (72%) and reaction time (3 h) was achieved. In addition, we also observed that if the ultrasound-assisted Rap–Stoermer reaction was performed in the absence of PEG-400, the desired products were not obtained in appreciable yields, which indicates that both the catalysis by PEG-400 and the ultrasonication together promoted this reaction. To establish the generality and applicability of this method, a wide variety of salicylaldehydes were subjected to the same set of conditions to furnish the corresponding 3-(2-benzofuroyl)carbazole derivatives. It was found that all the salicylaldehydes partners worked well. The reactions were generally complete within 4 h and the corresponding 3-(2-benzofuroyl)carbazole derivatives **3a**–**l** were produced in good yields of 60–72%, as shown in Table 1.

**Table 1:** Synthesis of 3-(2-benzofuroyl)carbazole derivatives (**3a**–**k**).

Entry	Compound **3**		Yield (%)^a^	mp (°C)

1	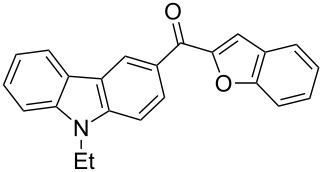	**3a**	72	102–103
2	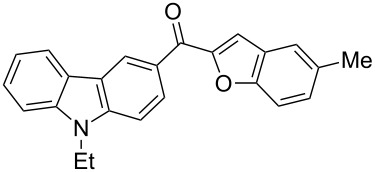	**3b**	68	157–159
3	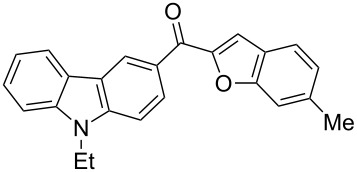	**3c**	62	144–145
4	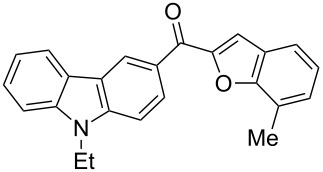	**3d**	63	120–122
5	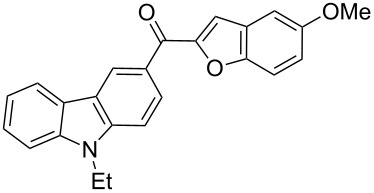	**3e**	66	129–130
6	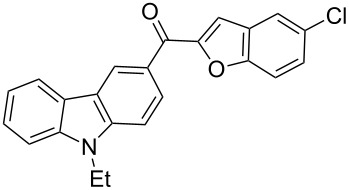	**3f**	65	135–136
7	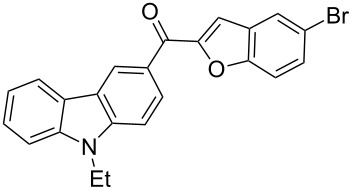	**3g**	67	119–120
8	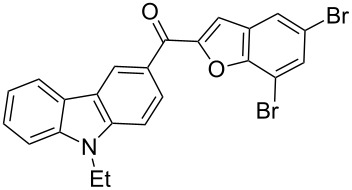	**3h**	69	228–230
9	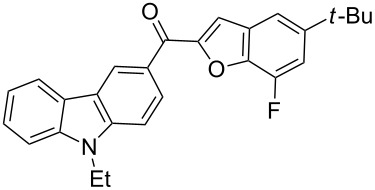	**3i**	62	<25
10	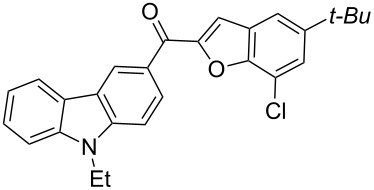	**3j**	60	<25
11	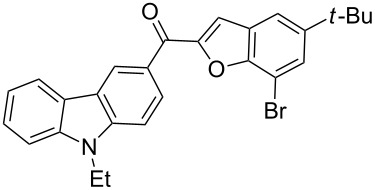	**3k**	61	<25

^a^ Isolated yield.

The results summarized in Table 1 indicated the scope and generality of the PEG-400-catalyzed, ultrasound-assisted Rap–Stoermer reaction with respect to various salicylaldehydes. Moreover, the presence of fluorine, chlorine, or bromine substituents (entries 6–11) is not problematic, thereby providing a potential handle for further functionalization (eg., Heck and Suzuki–Miyaura reactions) of the corresponding products **3f**–**k**. In the cases of entries 9–11, the *tert*-butyl-substituted products **3i**–**k** were isolated in pure form as semisolids by column chromatography over silica gel.

Next, we successfully extended our study towards the Rap–Stoermer reaction of 3,6-dichloroacetyl-*N*-ethyl-9*H*-carbazole (**4**) with these salicylaldehydes, under the same reaction conditions, to furnish the symmetrically substituted 3,6-bis(2-benzofuroyl)carbazoles **5a**–**k**, as shown in Table 2. The reaction of 3,6-dichloroacetyl-*N*-ethyl-9*H*-carbazole (**4**) proceeded smoothly and gave the desired compounds **5a**–**k** in 49–69% yields within 6 hours.

**Table 2:** Synthesis of 3,6-bis(2-benzofuroyl)carbazole derivatives (**5a**–**k**).

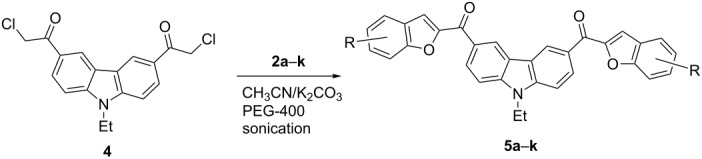				

Entry	Compound **5**		Yield (%)^a^	mp (°C)

1	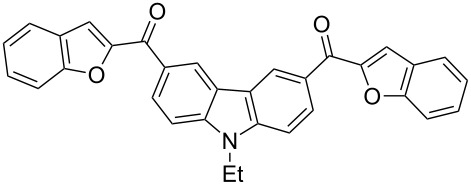	**5a**	63	213–214
2	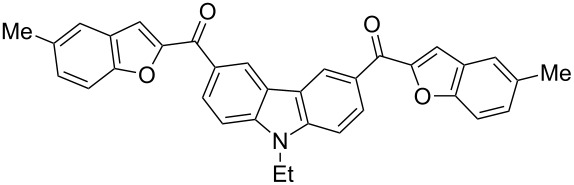	**5b**	58	202–204
3	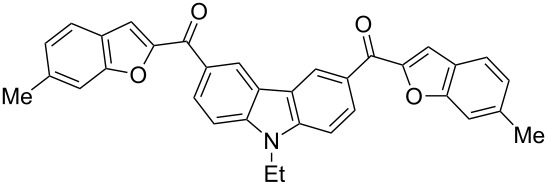	**5c**	51	191–192
4	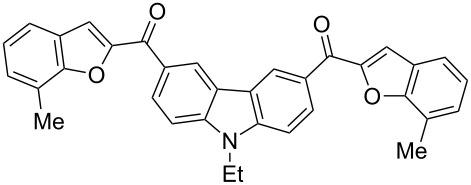	**5d**	56	206–208
5	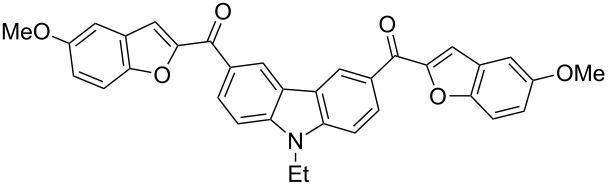	**5e**	52	201–203
6	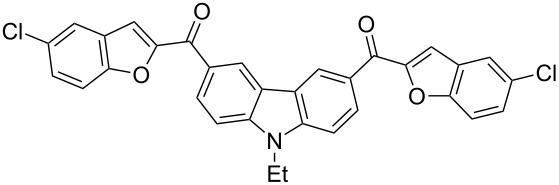	**5f**	69	245–247
7	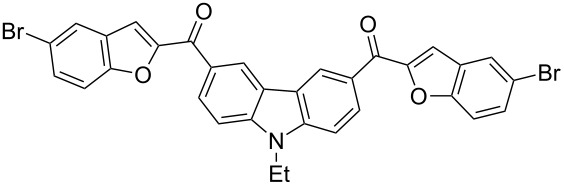	**5g**	65	259–260
8	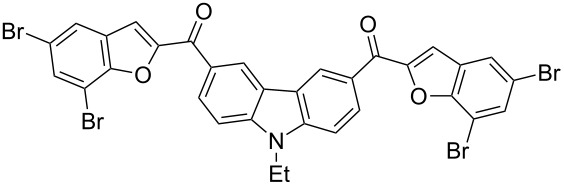	**5h**	59	293–295
9	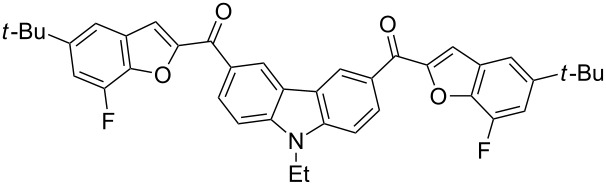	**5i**	54	200–202
10	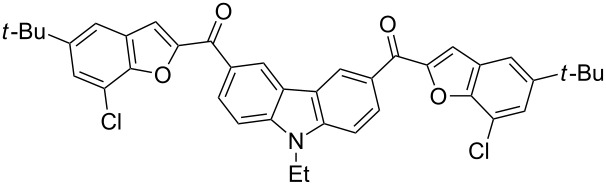	**5j**	52	174–176
11	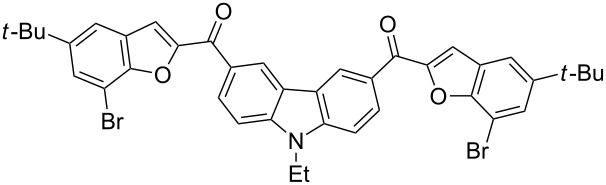	**5k**	49	161–162

^a^ Isolated yield.

**Scheme 2:**
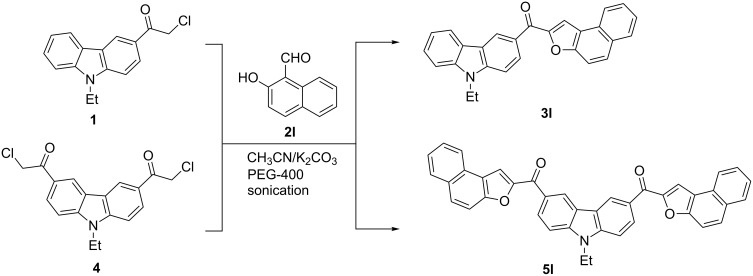
Synthesis of naphtho[2,1-*b*]furoyl-*N*-ethyl-9*H*-carbazole **3l** and **5l**.

Encouraged by these results, we also attempted the reaction of chloroacetylcarbazoles **1** and **4** with 2-hydroxy-1-naphthaldehyde (**2l**) with the aim of constructing novel naphthofuran derivatives. Interestingly, 2-hydroxy-1-napthaldehyde was equally amenable to the conditions and gave the corresponding 3-(2-naphtho[2,1-*b*]furoyl)-*N*-ethyl-9*H*-carbazole (**3l**) and 3,6-bis(2-naphtho[2,1-*b*]furoyl)-*N*-ethyl-9*H*-carbazole (**5l**) in good yields of 64% and 50%, respectively (Scheme 2).

All the newly synthesized compounds **3a**–**l** and **5a**–**l** were characterized by spectral analysis. All data were fully consistent with the assigned molecular structure (see Supporting Information File 1).

## Conclusion

In conclusion, we have achieved an efficient and straightforward method for the construction of a variety of novel benzofuroyl- as well as naphtho[2,1-*b*]furoyl-substituted carbazoles through an PEG-400-catalyzed and ultrasound-assisted Rap–Stoermer reaction. These molecules should allow us, in the future, to investigate structure–activity relationships in various biological tests or photonic applications. The ready availability of starting materials, mild reaction conditions, short reaction times, experimental simplicity and satisfactory yields contribute to the usefulness of this method. The possible biological activity of the described compounds possessing the benzofuran and carbazole skeletons remains to be studied. In addition, the products represent potentially useful synthetic building blocks in medicinal chemistry.

## Experimental

Melting points (uncorrected) were determined by using a WRS-1B melting-point apparatus. Ultrasonication was performed in a KQ-250B medical ultrasound cleaner at a frequency of 40 KHz and output power of 250 W (Built-in heating 30–80 °C, thermostatically adjustable). ^1^H NMR and ^13^C NMR spectra were recorded on a Bruker AVANCE NMR spectrometer with CDCl_3_ or DMSO-*d*_6_ as the solvent. The reported chemical shifts (δ values) are given in parts per million downfield from tetramethylsilane (TMS) as the internal standard. HRMS (ESI) data were acquired on a Bruker Custom micrOTOF-Q 125 high-resolution mass spectrometer. The progress of reactions was monitored by thin-layer chromatography (TLC) on silica gel GF254 with EtOAc/PE as eluent. Petroleum ether (PE) refers to the fraction that boils in the range of 60–90 °C.

**General procedure for the preparation of 3-(2-benzofuroyl)-*N*-ethyl-9*H*-carbazoles 3a**–**l.** To a stirred solution of 3-chloroacetyl-9-ethyl-9*H*-carbazole (**1**) (136 mg, 0.5 mmol) in acetonitrile (4 mL), the required salicylaldehydes **2a**–**k** or 2-hydroxy-1-naphthaldehyde (**2l**) (0.55 mmol), potassium carbonate (138 mg, 1 mmol) and PEG-400 (98 mg, 0.25 mmol) were added. The resulting mixture was sonicated at 80 °C for 2–4 hours. After the reaction was complete (TLC), the mixture was cooled to room temperature, poured into 5 mL of water and filtered to give the crude product, which was then purified by silica gel column chromatography with EtOAc/PE (1:6) as eluent. The melting points and yields of all the compounds are summarized in Table 1 and the spectral and analytical data are given in Supporting Information File 1.

**General procedure for the preparation of 3,6-bis(benzofuroyl)-*N*-ethyl-9*H*-carbazoles 5a**–**l.** To a stirred solution of 3,6-dichloroacetyl-9-ethyl-9*H*-carbazole (**4**) (174 mg, 0.5 mmol) in acetonitrile (4 mL), the required salicylaldehydes or 2-hydroxy-1-naphthaldehyde (1.1 mmol), potassium carbonate (276 mg, 2 mmol) and PEG-400 (98 mg, 0.25 mmol) were added. The resulting mixture was sonicated at 80 °C for 3–6 hours. After the reaction was complete (TLC), the mixture was cooled to room temperature, poured into water and filtered to give the crude product, which was then purified by silica gel column chromatography with EtOAc/PE (1:6) as eluent. The melting points and yields of all the compounds are summarized in Table 2 and the spectral and analytical data are given in Supporting Information File 1.

## Supporting Information

File 1 Characterization data of the title compounds and NMR and HRMS spectra.
