# Supplementary material for: A novel and facile synthesis of 3-(2-benzofuroyl)- and 3,6-bis(2-benzofuroyl)carbazole derivatives
Source: Beilstein J Org Chem. 2011 Nov 17;7:1533–40. doi: 10.3762/bjoc.7.180 (PMC3252855; doi:10.3762/bjoc.7.180)
Supplement: File 1 — Characterization data of the title compounds and NMR and HRMS spectra. [file Beilstein_J_Org_Chem-07-1533-s001.pdf]

Supporting Information

for

A novel and facile synthesis of 3-(2-benzofuroyl)- and 3,6-bis(2-benzofuroyl)carbazole derivatives

Wentao Gao*, Meiru Zheng, and Yang Li

Address: Institute of Superfine Chemicals, Bohai University, Jinzhou 121000, China

Email: Wentao Gao - bhuzh@163.com

* Corresponding author

Characterization data of the title compounds and NMR and HRMS spectra

Characterization data for products 3a–l

3-(2-Benzofuroyl)-*N*-ethyl-9*H*-carbazole (3a). Yellow solid. IR (KBr) v/cm^{-1}: 3068, 2972, 2938, 1638, 1591, 1436, 1231, 1062, 809, 747; ^{1}H NMR (300 MHz, $CDCl_3$): δ 1.51 (t, *J* = 7.2 Hz, 3H, CH_3CH_2), 4.46 (q, *J* = 7.2 Hz, 2H, CH_3CH_2), 7.36 (d, *J* = 8.0 Hz, 1H, ArH), 7.38 (d, *J* = 8.0 Hz, 1H, ArH), 7.48-7.55 (m, 4H, ArH), 7.61 (s, 1H, furan-H), 7.70 (d, *J* = 8.2 Hz, 1H, ArH), 7.78 (d, *J* = 7.8 Hz, 1H, ArH), 8.19 (d, *J* = 7.8 Hz, 1H, ArH), 8.29 (dd, *J* = 8.7, 1.50 Hz, 1H, ArH), 8.93 (d, *J* = 1.3 Hz, 1H, ArH); HRMS–ESI: (*m/z*) $[M + Na]^+$ calcd for $C_{23}H_{17}NNaO_2$: 362.1151; found: 362.1150.

3-[2-(5-Methylbenzofuroyl)]-*N*-ethyl-9*H*-carbazole (3b). Yellow solid. IR (KBr) v/cm^{-1}: 3065, 2963, 2915, 1640, 1591, 1432, 1022, 816, 775; ^{1}H NMR (600 MHz, $CDCl_3$): δ 1.49 (t, *J* = 7.2 Hz, 3H, CH_3CH_2), 2.54 (s, 3H, CH_3), 4.42 (q, *J* = 7.2 Hz, 2H, CH_3CH_2), 7.17 (d, *J* = 7.8 Hz, 1H, ArH), 7.32 (t, *J* = 7.8 Hz, 1H, ArH), 7.47-7.50 (m, 4H, ArH), 7.53 (d, *J* = 7.2 Hz, 1H, ArH), 7.54 (s, 1H, furan-H), 7.62 (d, *J* = 7.8 Hz, 1H, ArH), 8.26 (dd, *J* = 8.4, 1.8 Hz, 1H, ArH), 8.90 (d, *J* = 1.2 Hz, 1H, ArH); ^{13}C NMR (150 MHz, $CDCl_3$): δ 13.84, 22.09, 37.89, 108.21, 109.03, 112.49, 115.75, 120.04, 120.84, 122.56, 122.79, 123.29, 124.78, 125.56, 126.53, 127.76, 128.42, 138.75, 140.68, 142.74, 152.75, 156.41, 183.89; Anal. calcd for $C_{24}H_{19}NO_2$: C, 81.56; H, 5.42; N, 3.96; found: C, 81.42; H, 5.70; N, 3.75.

3-[2-(6-Methylbenzofuroyl)]-*N*-ethyl-9*H*-carbazole (3c). Yellow solid. IR (KBr) v/cm^{-1}: 3071, 2962, 2911, 1641, 1590, 1432, 1022, 816, 776; ^{1}H NMR (600 MHz,

$CDCl_3$): δ 1.49 (t, J = 7.2 Hz, 3H, CH_3CH_2), 2.48 (s, 3H, CH_3), 4.43 (q, J = 7.2 Hz, 2H, CH_3CH_2), 7.30-7.33 (m, 2H, ArH), 7.47 (d, J = 7.8 Hz, 1H, ArH), 7.49 (d, J = 8.4 Hz, 1H, ArH), 7.51 (s, 1H, furan-H), 7.53 (d, J = 7.8 Hz, 2H, ArH), 7.56 (d, J = 8.4 Hz, 1H, ArH), 8.18 (d, J = 7.8 Hz, 1H, ArH), 8.27 (dd, J = 8.4, 1.8 Hz, 1H, ArH), 8.90 (s, 1H, ArH); ^{13}C NMR (150 MHz, $CDCl_3$): δ 13.83, 21.34, 37.89, 108.21, 109.03, 112.04, 115.34, 120.05, 120.83, 122.58, 122.80, 123.52, 123.35, 126.54, 127.32, 127.80, 128.33, 129.41, 133.40, 140.68, 142.76, 153.26, 154.39, 183.91; Anal. calcd for $C_{24}H_{19}NO_2$: C, 81.56; H, 5.42; N, 3.96; found: C, 81.38; H, 5.43; N, 3.82.

3-[2-(7-Methylbenzofuroyl)]-*N*-ethyl-9*H*-carbazole (3d). Yellow solid. IR (KBr) v/cm^{-1}: 3069, 2966, 2916, 1641, 1590, 1432, 1021, 815, 779; ^1H NMR (600 MHz, $CDCl_3$): δ 1.48 (t, J = 7.2 Hz, 3H, CH_3CH_2), 2.53 (s, 3H, CH_3), 4.42 (q, J = 7.2 Hz, 2H, CH_3CH_2), 7.16 (d, J = 7.8 Hz, 1H, ArH), 7.31 (d, J = 7.8 Hz, 1H, ArH), 7.45-7.53 (m, 5H, ArH and furan-H), 7.61 (d, J = 7.8 Hz, 1H, ArH), 8.17 (d, J = 7.8 Hz, 1H, ArH), 8.25 (d, J = 8.4 Hz, 1H, ArH), 8.89 (s, 1H, ArH); ^{13}C NMR (150 MHz, $CDCl_3$): δ 13.81, 22.07, 37.85, 108.18, 109.00, 112.45, 115.73, 120.01, 120.81, 122.54, 122.76, 123.26, 124.76, 125.54, 126.51, 127.73, 128.38, 138.72, 140.65, 142.70, 152.73, 156.39, 183.84; Anal. calcd for $C_{24}H_{19}NO_2$: C, 81.56; H, 5.42; N, 3.96; found: C, 81.76; H, 5.24; N, 3.75.

3-[2-(5-Methoxybenzofuroyl)]-*N*-ethyl-9*H*-carbazole (3e). Yellow solid. IR (KBr) v/cm^{-1}: 3071, 2962, 2911, 1641, 1590, 1432, 1036, 816, 776; ^1H NMR (600 MHz, $CDCl_3$): δ 1.50 (t, J = 7.2 Hz, 3H, CH_3CH_2), 3.89 (s, 3H, OCH_3), 4.44 (q, J = 7.2 Hz, 2H, CH_3CH_2), 7.12 (dd, J = 9.0, 2.4 Hz, 1H, ArH), 7.15 (d, J = 2.4 Hz, 1H, ArH), 7.32 (t, J =

7.2 Hz, 1H, ArH), 7.48 (d, *J* = 8.4, 1H, ArH), 7.50 (d, *J* = 9.0 Hz, 1H, ArH), 7.57 (d, *J* = 9.0 Hz, 1H, ArH), 7.51-7.55 (m, 2H, ArH and furan-H), 8.19 (d, *J* = 7.8 Hz, 1H, ArH), 8.27 (dd, *J* = 8.4, 1.2 Hz, 1H, ArH), 8.91 (s, 1H, ArH); ^{13}C NMR (150 MHz, $CDCl_3$): δ 13.84, 37.90, 55.89, 103.93, 108.23, 109.04, 113.17, 115.50, 117.85, 120.07, 120.84, 122.80, 123.26, 123.39, 126.56, 127.70, 127.83, 128.26, 140.68, 142.79, 151.08, 153.89, 156.59, 183.73; Anal. calcd for $C_{24}H_{19}NO_3$: C, 78.03; H, 5.18; N, 3.79; found: C, 77.82; H, 5.36; N, 3.57.

3-[2-(5-Chlorobenzofuroyl)]-*N*-ethyl-9*H*-carbazole (3f). Yellow solid. IR (KBr) v/cm^{-1}: 3091, 2983, 2940, 1641, 1591, 1435, 1232, 1062, 1022, 816, 773; ^{1}H NMR (300 MHz, $CDCl_3$): δ 1.50 (t, *J* = 7.2 Hz, 3H, CH_3CH_2), 4.47 (q, *J* = 7.2 Hz, 2H, CH_3CH_2), 7.26 (d, *J* = 7.8 Hz, 1H, ArH), 7.44-7.58 (m, 5H, ArH), 7.63 (s, 1H, furan-H), 7.79 (d, *J* = 2.0 Hz, 1H, ArH), 8.24 (d, *J* = 7.8 Hz, 1H, ArH), 8.29 (dd, *J* = 8.7, 1.6 Hz, 1H, ArH), 8.90 (d, *J* = 1.4 Hz, 1H, ArH); Anal. calcd for $C_{23}H_{16}ClNO_2$: C, 73.90; H, 4.31; N, 3.75; found: C, 74.07; H, 4.24; N, 3.73.

3-[2-(5-Bromobenzofuroyl)]-*N*-ethyl-9*H*-carbazole (3g). Yellow solid. IR (KBr) v/cm^{-1}: 3063, 2982, 2937, 1642, 1591, 1434, 1232, 1022, 970, 814, 772, 622; ^{1}H NMR (300 MHz, $CDCl_3$): δ 1.49 (t, *J* = 7.2 Hz, 3H, CH_3CH_2), 4.48 (q, *J* = 7.2 Hz, 2H, CH_3CH_2), 7.41-7.52 (m, 4H, ArH), 7.58-7.64 (m, 3H, ArH), 7.88 (s, 1H, furan-H), 8.17 (d, *J* = 7.8 Hz, 1H, ArH), 8.25 (dd, *J* = 8.6, 1.5 Hz, 1H, ArH), 8.89 (d, *J* = 1.4 Hz, 1H, ArH); Anal. calcd for $C_{23}H_{16}BrNO_2$: C, 66.04; H, 3.86; N, 3.35; found: C, 66.26; H, 3.72; N, 3.25.

3-[2-(5,7-Dibromobenzofuroyl)]-*N*-ethyl-9*H*-carbazole (3h). Yellow solid. IR (KBr) v/cm^{-1}: 3069, 2966, 2916, 1641, 1590, 1432, 1021, 815, 779, 662; ^{1}H NMR (600 MHz, $CDCl_3$): *δ* 1.37 (t, *J* = 7.2 Hz, 3H, CH_3CH_2), 4.55 (q, *J* = 7.2 Hz, 2H, CH_3CH_2), 7.32 (t, *J* = 7.2 Hz, 1H, ArH), 7.56 (t, *J* = 7.8 Hz, 1H, ArH), 7.73 (d, *J* = 8.4 Hz, 1H, ArH), 7.83 (d, *J* = 8.4 Hz, 1H, ArH), 7.88 (s, 1H, furan-H), 8.04 (d, *J* = 1.8 Hz, 1H, ArH), 8.12 (d, *J* = 1.8 Hz, 1H, ArH), 8.19 (dd, *J* = 8.4, 1.2 Hz, 1H, ArH), 8.32 (d, *J* = 7.8 Hz, 1H, ArH), 8.99 (s, 1H, ArH); ^{13}C NMR (150 MHz, DMSO): *δ* 13.82, 37.44, 105.35, 109.44, 109.96, 115.59, 116.42, 120.16, 121.04, 122.15, 122.53, 123.40, 125.64, 126.86, 127.19, 127.46, 129.95, 132.35, 140.46, 142.65, 151.38, 153.64, 182.16; Anal. calcd for $C_{23}H_{15}Br_2NO_2$: C, 55.56; H, 3.04; N, 2.82; found: C, 55.28; H, 3.09; N, 2.58.

3-[2-(5-*tert*-Butyl-7-fluorobenzofuroyl)]-*N*-ethyl-9*H*-carbazole (3i). Yellow solid. IR (KBr) v/cm^{-1}: 3065, 2972, 2881, 1641, 1592, 1435, 1231, 1192, 1024, 815, 778; ^{1}H NMR (300 MHz, $CDCl_3$): *δ* 1.40 (s, 9H, *t*-butyl), 1.50 (t, *J* = 7.2 Hz, 3H, CH_3CH_2), 4.44 (q, *J* = 7.2 Hz, 2H, CH_3CH_2), 7.29-7.35 (m, 2H, ArH), 7.46-7.54 (m, 4H, ArH), 7.60 (d, *J* = 2.7 Hz, 1H, ArH), 8.18 (d, *J* = 7.7 Hz, 1H, ArH), 8.32 (dd, *J* = 8.6, 1.4 Hz, 1H, ArH), 8.97 (s, 1H, ArH); HRMS–ESI: (*m/z*) $[M + Na]^+$ calcd for $C_{27}H_{24}FNNaO_2$: 436.1683; found: 436.1688.

3-[2-(5-*tert*-Butyl-7-chlorobenzofuroyl)]-*N*-ethyl-9*H*-carbazole (3j). Yellow solid. IR (KBr) v/cm^{-1}: 3058, 2964, 2870, 1640, 1589, 1430, 1229, 1084, 1022, 815, 777; ^{1}H NMR (300 MHz, $CDCl_3$): *δ* 1.42 (s, 9H, *t*-butyl), 1.50 (t, *J* = 7.2 Hz, 3H, CH_3CH_2), 4.45 (q, *J* = 7.2 Hz, 2H, CH_3CH_2), 7.32-7.41 (m, 1H, ArH), 7.47-7.58 (m, 5H, ArH), 7.64 (s,

1H, furan-H), 8.20 (d, J = 7.7 Hz, 1H, ArH), 8.38 (d, J = 8.6 Hz, 1H, ArH), 9.08 (s, 1H, ArH); Anal. calcd for $C_{27}H_{24}ClNO_2$: C, 75.43; H, 5.63; N, 3.26 found: C, 75.21; H, 5.52; N, 3.34.

3-[2-(7-Bromo-5-*tert*-butylbenzofuroyl)]-*N*-ethyl-9*H*-carbazole (3k). Yellow solid. IR (KBr) ν/cm^{-1}: 3070, 2963, 2869, 1640, 1587, 1431, 1228, 1022, 815, 780, 658; ^{1}H NMR (300 MHz, $CDCl_3$): δ 1.24 (t, J = 7.2 Hz, 3H, CH_3CH$_2$), 1.40 (s, 9H, *t*-butyl), 3.73 (q, J = 7.2 Hz, 2H, CH$_3$CH_2), 7.29-7.34 (m, 2H, ArH), 7.45-7.55 (m, 3H, ArH), 7.66 (s, 1H, furan-H), 7.71 (s, 1H, ArH), 8.19 (d, J = 7.6 Hz, 1H, ArH), 8.37 (d, J = 8.6 Hz, 1H, ArH), 9.12 (s, 1H, ArH); Anal. calcd for $C_{27}H_{24}BrNO_2$: C, 68.36; H, 5.10; N, 2.95; found: C, 68.22; H, 5.34; N, 2.87.

3-(2-Naphtho[2,1-*b*]furoyl)-*N*-ethyl-9*H*-carbazole (3l). Brown solid. IR (KBr) ν/cm^{-1}: 3071, 2972, 2931, 1642, 1590, 1436, 1232, 1065, 808, 765; ^{1}H NMR (300 MHz, $CDCl_3$): δ 1.52 (t, J = 7.2 Hz, 3H, CH_3CH$_2$), 4.48 (q, J = 7.2 Hz, 2H, CH$_3$CH_2), 7.32-7.37 (m, 1H, ArH), 7.49 (d, J = 8.0 Hz, 1H, ArH), 7.53-7.60 (m, 3H, ArH), 7.64-7.69 (m, 1H, ArH), 7.81 (d, J = 8.8 Hz, 1H, ArH), 7.94 (d, J = 9.0 Hz, 1H, ArH), 8.01 (d, J = 8.0 Hz, 1H, ArH), 8.10 (d, J = 0.5 Hz, 1H, ArH), 8.22 (d, J = 7.8 Hz, 2H, ArH), 8.34 (dd, J = 8.6, 1.7 Hz, 1H, ArH), 8.98 (d, J = 1.4 Hz, 1H, ArH); HRMS–ESI: (*m/z*) $[M + Na]^+$ calcd for $C_{27}H_{19}NNaO_2$: 412.1308. Found: 412.1309.

Characterization data for products 5a–l

3,6-Bis(benzofuroyl)-*N*-ethyl-9*H*-carbazole (5a). Yellow solid. IR (KBr) v/cm^{-1}: 3071, 2972, 2938, 1643, 1591, 1436, 1062, 809, 747; ^{1}H NMR (600 MHz, $CDCl_3$): δ 1.55 (t, J = 7.2 Hz, 3H, CH_3CH_2), 4.50 (q, J = 7.2 Hz, 2H, CH_3CH_2), 7.36 (t, J = 7.8 Hz, 2H, ArH), 7.52 (t, J = 7.8 Hz, 2H, ArH), 7.59 (d, J = 9.0 Hz, 2H, ArH), 7.62 (s, 2H, furan-H), 7.69 (d, J = 8.4 Hz, 2H, ArH), 7.77 (d, J = 7.8 Hz, 2H, ArH), 8.33 (dd, J = 8.4, 1.2 Hz, 2H, ArH), 8.97 (d, J = 1.2 Hz, 2H, ArH); Anal. calcd for $C_{32}H_{21}NO_4$: C, 79.49; H, 4.38; N, 2.90; found: C, 79.40; H, 4.53; N, 2.77.

3,6-Bis[2-(5-methylbenzofuroyl)]-*N*-ethyl-9*H*-carbazole (5b). Yellow solid. IR (KBr) v/cm^{-1}: 3071, 2962, 2911, 1641, 1590, 1432, 1022, 816, 776; ^{1}H NMR (600 MHz, $CDCl_3$): δ 1.57 (t, J = 7.2 Hz, 3H, CH_3CH_2), 2.53 (s, 6H, CH_3), 4.50 (q, J = 7.2 Hz, 2H, CH_3CH_2), 7.18 (d, J = 8.4 Hz, 2H, ArH), 7.48 (s, 2H, ArH), 7.57 (d, J = 8.4 Hz, 2H, ArH), 7.58 (s, 2H, furan-H), 7.63 (d, J = 7.8 Hz, 2H, ArH), 8.32 (dd, J = 9.0, 1.8 Hz, 2H, ArH), 8.97 (s, 2H, ArH); ^{13}C NMR (150 MHz, $CDCl_3$): δ 13.91, 22.10, 38.29, 108.91, 112.51, 116.14, 122.68, 123.04, 123.49, 124.72, 125.68, 128.50, 129.56, 139.02, 143.43, 152.51, 156.50, 183.70; HRMS–ESI: (*m/z*) [M + Na]$^+$ calcd for $C_{34}H_{25}NNaO_4$: 534.1676; found: 534.1678.

3,6-Bis[2-(6-methylbenzofuroyl)]-*N*-ethyl-9*H*-carbazole (5c). Yellow solid. IR (KBr) v/cm^{-1}: 3069, 2966, 2916, 1641, 1590, 1432, 1021, 815, 779; ^{1}H NMR (600 MHz, $CDCl_3$): δ 1.54 (t, J = 7.2 Hz, 3H, CH_3CH_2), 2.48 (s, 6H, CH_3), 4.51 (q, J = 7.2 Hz, 2H,

CH_3CH_2), 7.31 (d, *J* = 9.0 Hz, 2H, ArH), 7.53-7.58 (m, 8H, ArH and furan-H), 8.32 (d, *J* = 9.0 Hz, 2H, ArH), 8.97 (s, 2H, ArH); ^{13}C NMR (150 MHz, $CDCl_3$): δ 13.91, 21.34, 38.29, 108.94, 112.06, 115.75, 122.70, 123.05, 123.55, 127.25, 128.55, 129.50, 129.65, 133.53, 143.47, 152.99, 154.48, 183.74; HRMS–ESI: (*m/z*) $[M + Na]^+$ calcd for $C_{34}H_{25}NNaO_4$: 534.1676; found: 534.1672.

3,6-Bis[2-(7-methylbenzofuroyl)]-*N*-ethyl-9*H*-carbazole (5d). Yellow solid. IR (KBr) v/cm^{-1}: 3065, 2963, 2915, 1640, 1591, 1432, 1022, 816, 775; ^1H NMR (600 MHz, $CDCl_3$): δ 1.55 (t, *J* = 7.2 Hz, 3H, CH_3CH_2), 2.48 (s, 6H, CH_3), 4.50 (q, *J* = 7.2 Hz, 2H, CH_3CH_2), 7.32 (d, *J* = 9.0 Hz, 2H, ArH), 7.54-7.59 (m, 8H, ArH and furan-H), 8.33 (d, *J* = 9.0 Hz, 2H, ArH), 8.97 (d, *J* = 1.2 Hz, 2H, ArH); HRMS–ESI: (*m/z*) $[M + Na]^+$ calcd for $C_{34}H_{25}NNaO_4$: 534.1676; found: 534.1700.

3,6-Bis[2-(5-methoxybenzofuroyl)]-*N*-ethyl-9*H*-carbazole (5e). Yellow solid. IR (KBr) v/cm^{-1}: 3071, 2962, 2911, 1641, 1590, 1432, 1036, 816, 776; ^1H NMR (600 MHz, $CDCl_3$): δ 1.49 (t, *J* = 7.2 Hz, 3H, CH_3CH_2), 3.88 (s, 6H, OCH_3), 4.46 (q, *J* = 7.2 Hz, 2H, CH_3CH_2), 7.13 (dd, *J* = 9.0, 2.4 Hz, 2H, ArH), 7.15 (d, *J* = 2.4 Hz, 2H, ArH), 7.56-7.58 (m, 6H, ArH and furan-H), 8.38 (dd, *J* = 8.4, 1.2 Hz, 2H, ArH), 8.89 (s, 2H, ArH); ^{13}C NMR (150 MHz, $CDCl_3$): δ 13.91, 38.29, 55.87, 103.93, 108.94, 113.20, 115.93, 118.19, 123.04, 123.56, 127.63, 128.57, 129.41, 143.48, 151.12, 153.56, 156.65, 183.54; HRMS–ESI: (*m/z*) $[M + Na]^+$ calcd for $C_{34}H_{25}NNaO_6$: 566.1574; found: 566.1570.

3,6-Bis[2-(5-chlorobenzofuroyl)]-*N*-ethyl-9*H*-carbazole (5f). Yellow solid. IR (KBr) ν/cm^{-1}: 3072, 2983, 2940, 1641, 1591, 1436, 1062, 1022, 816, 783; ^{1}H NMR (600 MHz, $CDCl_3$): *δ* 1.56 (t, *J* = 7.2 Hz, 3H, CH_3CH_2), 4.52 (q, *J* = 7.2 Hz, 2H, CH_3CH_2), 7.21 (d, *J* = 8.4 Hz, 2H, ArH), 7.46 (s, 2H, ArH), 7.57 (s, 2H, furan-H), 7.62 (d, *J* = 8.4 Hz, 2H, ArH), 7.66 (d, *J* = 7.8 Hz, 2H, ArH), 8.35 (dd, *J* = 9.0, 1.8 Hz, 2H, ArH), 8.96 (s, 2H, ArH); HRMS–ESI: (*m/z*) $[M + Na]^+$ calcd for $C_{32}H_{19}{}^{35}Cl_2NNaO_4$: 574.0583; found: 574.0589.

3,6-Bis[2-(5-bromobenzofuroyl)]-*N*-ethyl-9*H*-carbazole (5g). Yellow solid. IR (KBr) ν/cm^{-1}: 3063, 2982, 2937, 1642, 1591, 1434, 1022, 814, 772, 622; ^{1}H NMR (600 MHz, $CDCl_3$): *δ* 1.56 (t, *J* = 7.2 Hz, 3H, CH_3CH_2), 4.51 (q, *J* = 7.2 Hz, 2H, CH_3CH_2), 7.56-7.61 (m, 8H, ArH and furan-H), 7.90 (d, *J* = 1.8 Hz, 2H, benzofuran-H), 8.33 (dd, *J* = 8.4, 1.2 Hz, 2H, ArH), 8.96 (s, 2H, ArH); Anal. calcd for $C_{32}H_{19}Br_2NO_4$: C, 59.93; H, 2.99; N, 2.18; found: C, 60.22; H, 2.79; N, 2.07.

3,6-Bis[2-(5,7-dibromobenzofuroyl)]-*N*-ethyl-9*H*-carbazole (5h). Yellow solid. IR (KBr) ν/cm^{-1}: 3069, 2966, 2916, 1641, 1590, 1432, 1021, 815, 779, 662; ^{1}H NMR (600 MHz, $CDCl_3$): *δ* 1.55 (t, *J* = 7.2 Hz, 3H, CH_3CH_2), 4.48 (q, *J* = 7.2 Hz, 2H, CH_3CH_2), 7.27 (d, *J* = 7.2 Hz, 2H, ArH), 7.51 (s, 2H, furan-H), 7.57 (d, *J* = 8.4 Hz, 2H, ArH), 7.63 (d, *J* = 7.2 Hz, 2H, ArH), 8.37 (q, *J* = 1.2 Hz, 2H, ArH), 9.14 (s, 2H, ArH); HRMS–ESI: (*m/z*) $[M + Na]^+$ calcd for $C_{32}H_{17}{}^{79}Br_4NNaO_4$: 817.7784; found: 817.7792. Anal. calcd for $C_{32}H_{17}Br_4NO_4$: C, 48.10; H, 2.14; N, 1.75; found: C, 48.34; H, 1.86; N, 1.76.

3,6-Bis[2-(5-*tert*-butyl-7-fluorobenzofuroyl)]-*N*-ethyl-9*H*-carbazole (5i). Yellow solid. IR (KBr) v/cm^{-1}: 3065, 2972, 2881, 1641, 1592, 1435, 1192, 1024, 815, 778; ^{1}H NMR (600 MHz, $CDCl_3$): δ 1.40 (s, 18H, *t*-butyl), 1.56 (t, J = 7.2 Hz, 3H, CH_3CH_2), 4.51 (q, J = 7.2 Hz, 2H, CH_3CH_2), 7.27-7.31 (m, 2H, ArH), 7.51 (s, 2H, furan-H), 7.58 (d, J = 8.4 Hz, 2H, ArH), 7.62 (d, J = 2.4 Hz, 2H, ArH), 8.36 (dd, J = 8.4, 1.2 Hz, 2H, ArH), 9.00 (s, 2H, ArH); ^{13}C NMR (150 MHz, $CDCl_3$): δ 13.91, 31.56, 35.04, 38.31, 109.10, 112.12, 112.23, 114.48, 115.71, 123.02, 123.73, 128.62, 129.07, 130.02, 141.22, 141.30, 143.58, 146.86, 148.52, 148.75, 153.99, 182.98; HRMS–ESI: (*m/z*) $[M + Na]^+$ calcd for $C_{40}H_{35}F_2NNaO_4$: 654.2426; found: 654.2424.

3,6-Bis[2-(5-*tert*-butyl-7-chlorobenzofuroyl)]-*N*-ethyl-9*H*-carbazole (5j). Yellow solid. IR (KBr) v/cm^{-1}: 3058, 2964, 2870, 1640, 1589, 1430, 1062, 1022, 815, 777; ^{1}H NMR (600 MHz, $CDCl_3$): δ 1.32 (s, 18H, *t*-butyl), 1.46 (t, J = 7.2 Hz, 3H, CH_3CH_2), 4.40 (q, J = 7.2 Hz, 2H, CH_3CH_2), 7.46 (d, J = 1.8 Hz, 2H, benzofuran-H), 7.49 (d, J = 8.4 Hz, 2H, carbazole-H), 7.55 (s, 2H, furan-H), 7.57 (d, J = 1.8 Hz, 2H, benzofuran-H), 8.29 (dd, J = 8.4, 1.2 Hz, 2H, carbazole-H), 8.96 (d, J = 0.6 Hz, 2H, carbazole-H); ^{13}C NMR (150 MHz, $CDCl_3$): δ 13.93, 31.58, 35.01, 38.27, 109.14, 115.83, 116.94, 117.76, 122.96, 123.79, 126.15, 128.64, 128.61, 128.96, 143.52, 148.52, 149.88, 153.79, 182.71; HRMS–ESI: (*m/z*) $[M + Na]^+$ calcd for $C_{40}H_{35}{}^{35}Cl_2NNaO_4$: 686.1835; found: 686.1835.

3,6-Bis[2-(5-*tert*-butyl-7-bromobenzofuroyl)]-*N*-ethyl-9*H*-carbazole (5k). Yellow solid. IR (KBr) v/cm^{-1}: 3070, 2963, 2869, 1640, 1587, 1431, 1022, 815, 780, 658; ^{1}H

NMR (600 MHz, $CDCl_3$): δ 1.41 (s, 18H, *t*-butyl), 1.56 (t, *J* = 7.2 Hz, 3H, CH_3CH_2), 4.52 (q, *J* = 7.2 Hz, 2H, CH_3CH_2), 7.60 (d, *J* = 9.0 Hz, 2H, carbazole-H), 7.68 (s, 2H, furan-H), 7.71 (s, 2H, benzofuran-H), 7.73 (s, 2H, benzofuran-H), 8.42 (dd, *J* = 8.4, 1.2 Hz, 2H, carbazole-H), 9.13 (d, *J* = 1.2 Hz, 2H, carbazole-H); ^{13}C NMR (150 MHz, $CDCl_3$): δ 13.97, 31.62, 35.01, 38.31, 104.34, 109.18, 115.91, 118.45, 123.06, 123.95, 127.89, 128.68, 129.00, 129.07, 143.58, 148.88, 151.31, 153.83, 182.64; HRMS–ESI: (*m/z*) $[M + Na]^+$ calcd for $C_{40}H_{35}{}^{79}Br_2NNaO_4$: 774.0825; found: 774.0814.

3,6-Bis(2-naphtho[2,1-*b*]furoyl)-*N*-ethyl-9*H*-carbazole (5l). Brown solid. IR (KBr) v/cm^{-1}: 3071, 2972, 2931, 1642, 1590, 1436, 1232, 1065, 808, 765; ^1H NMR (600 MHz, $CDCl_3$): δ 1.57 (t, *J* = 7.2 Hz, 3H, CH_3CH_2), 4.50 (q, *J* = 7.2 Hz, 2H, CH_3CH_2), 7.55 (t, *J* = 7.8 Hz, 2H, ArH), 7.59 (d, *J* = 8.4 Hz, 2H, ArH), 7.63 (t, *J* = 7.8 Hz, 2H, ArH), 7.77 (d, *J* = 9.0 Hz, 2H, ArH), 7.89 (d, *J* = 9.0 Hz, 2H, ArH), 7.97 (d, *J* = 8.4 Hz, 2H, ArH), 8.10 (s, 2H, furan-H), 8.21 (d, *J* = 7.8 Hz, 2H, ArH), 8.38 (dd, *J* = 8.4, 1.2 Hz, 2H, ArH), 9.04 (s, 2H, ArH); ^{13}C NMR (150 MHz, $CDCl_3$): δ 13.91, 38.25, 108.97, 112.86, 114.87, 122.98, 123.05, 123.47, 123.53, 125.46, 127.33, 128.18, 128.54, 129.04, 129.52, 129.68, 130.54, 143.40, 152.59, 154.34, 183.08 ppm; Anal. calcd for $C_{40}H_{25}NO_4$: C, 82.32; H, 4.32; N, 2.40; found: C, 82.68; H, 4.25; N, 2.57.

Spectra for all the synthesised compounds 3a–5l

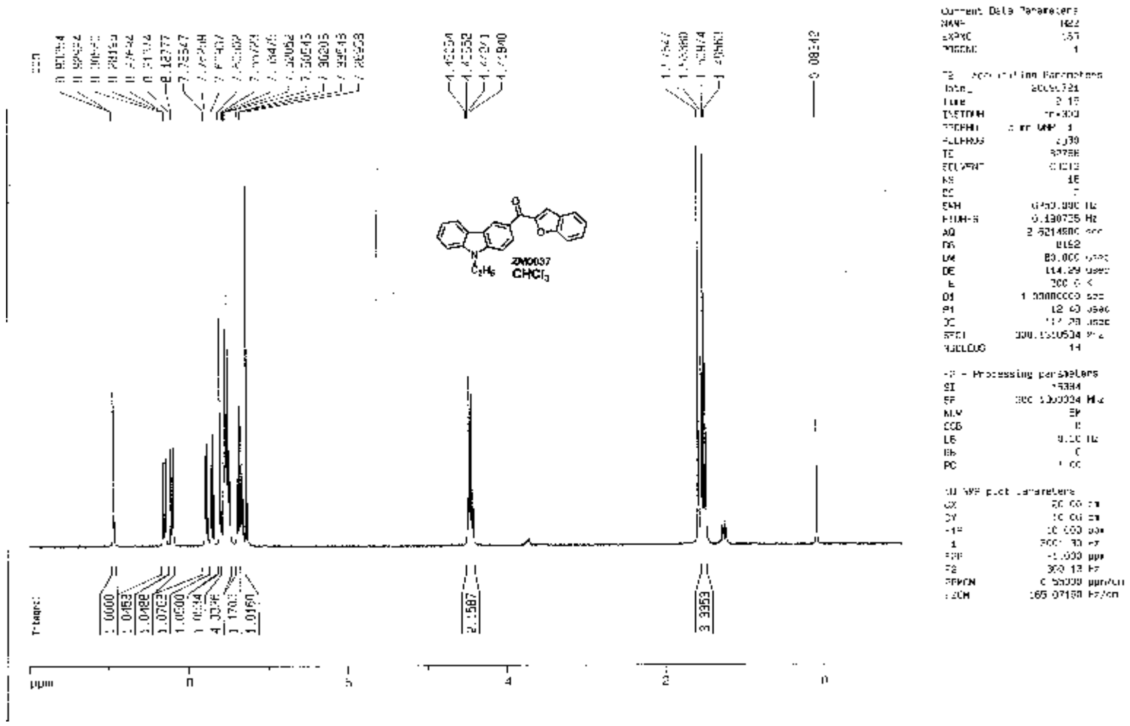

Figure S1: ^{1}H NMR spectrum of **3a**.

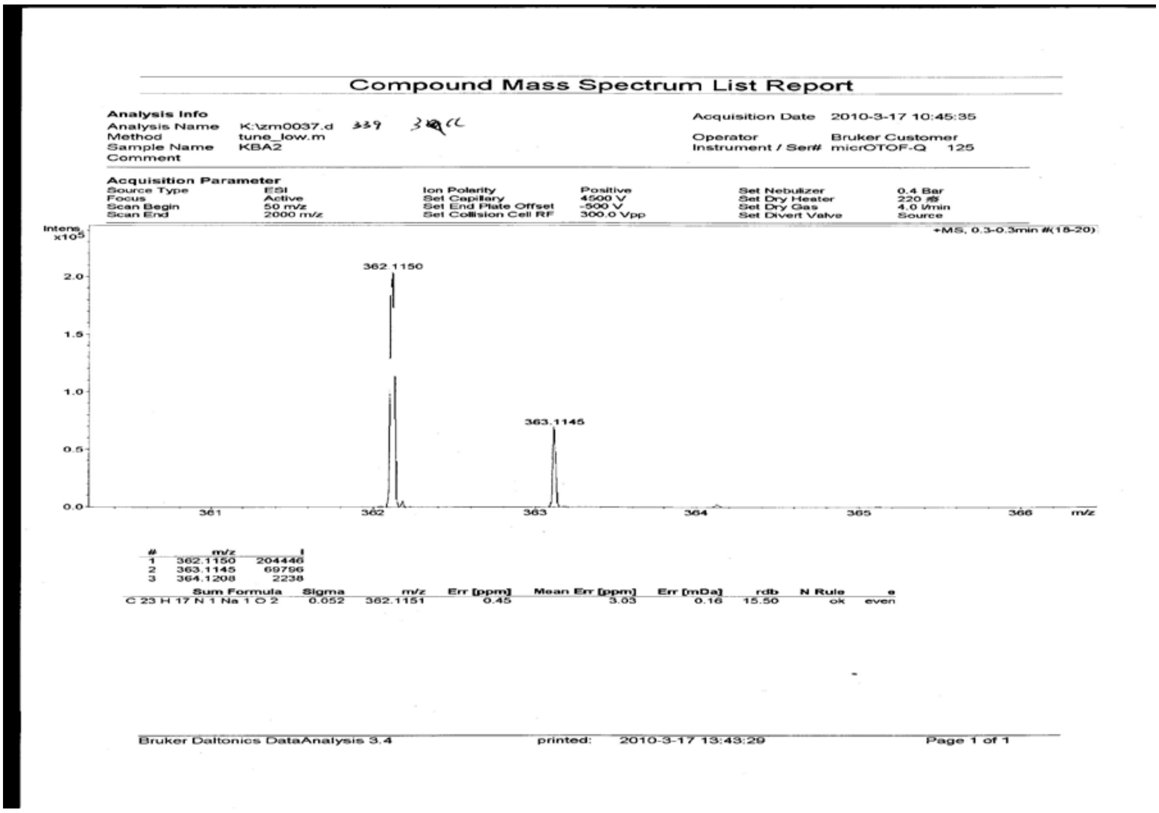

Figure S2: HRMS spectrum of **3a**.

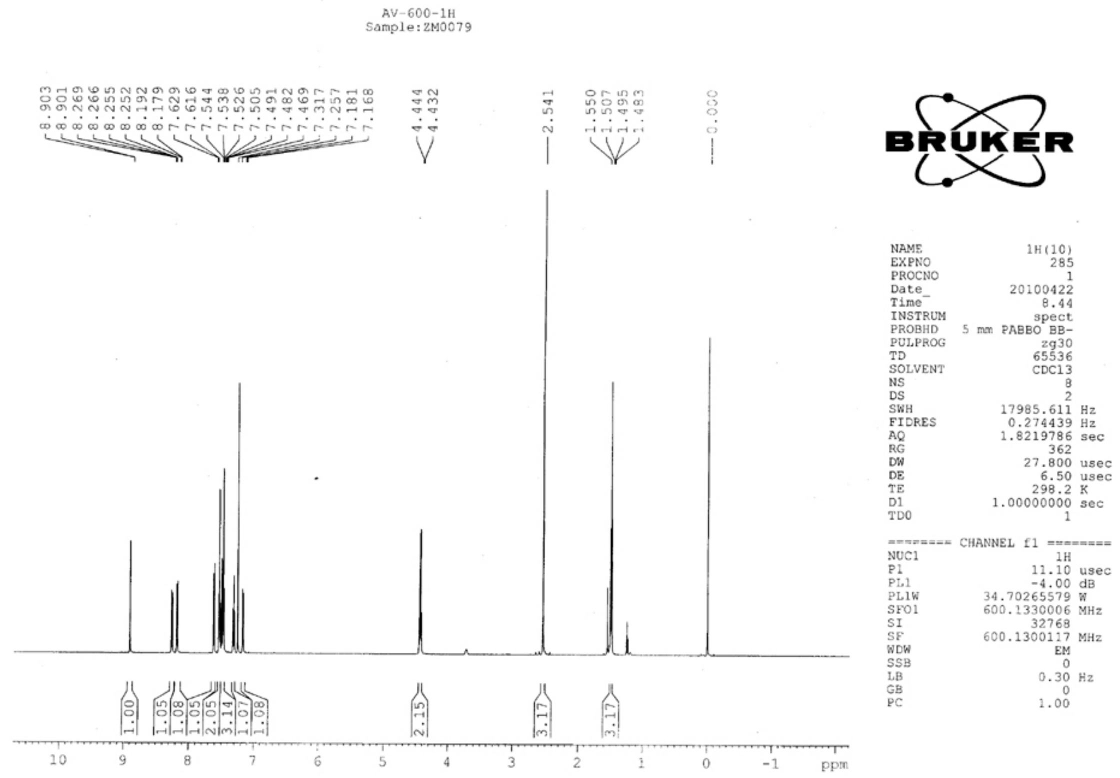

Figure S3: ^{1}H NMR spectrum of **3b**.

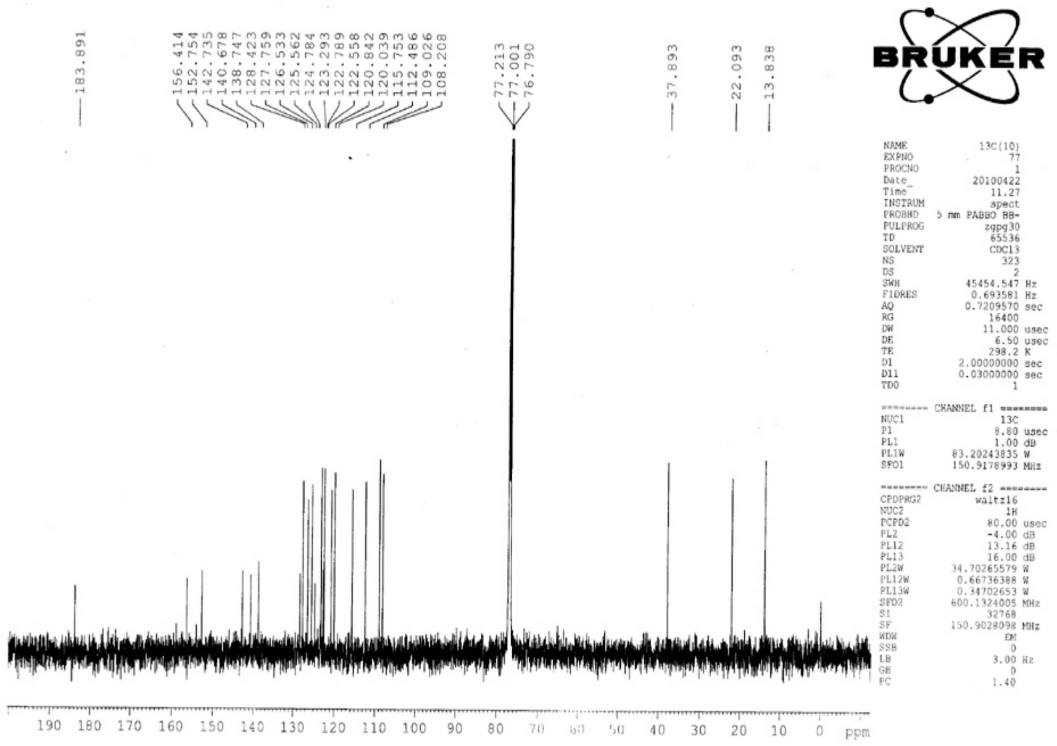

Figure S4: ^{13}C NMR spectrum of **3b**.

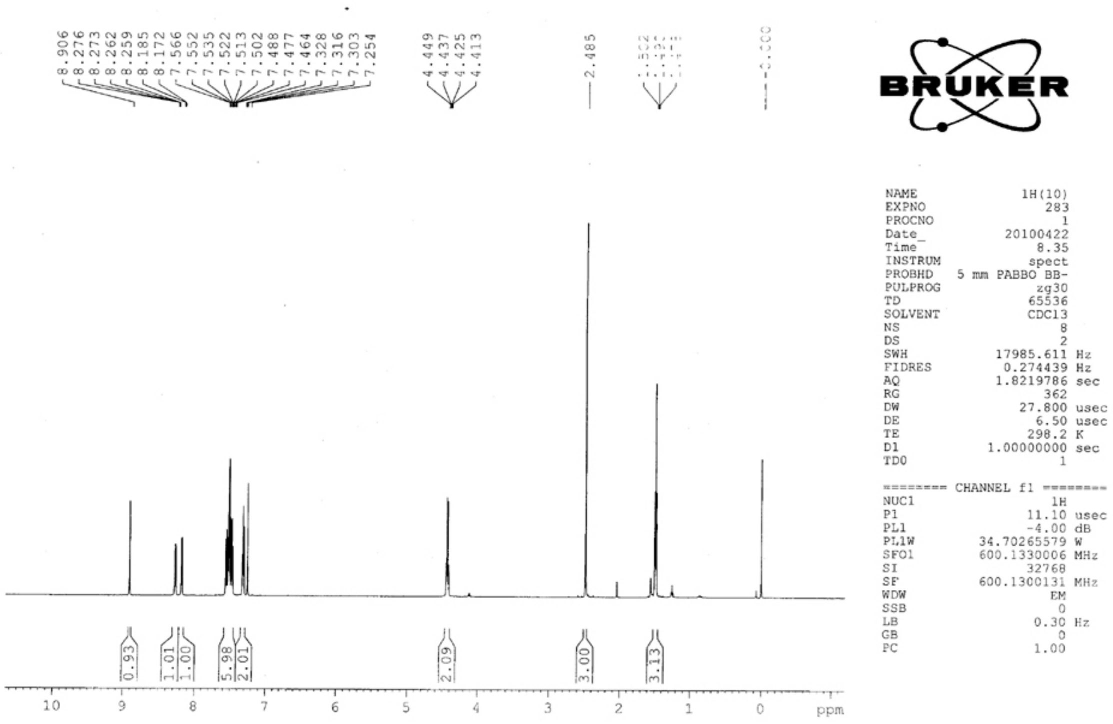

Figure S5: ^{1}H NMR spectrum of **3c**.

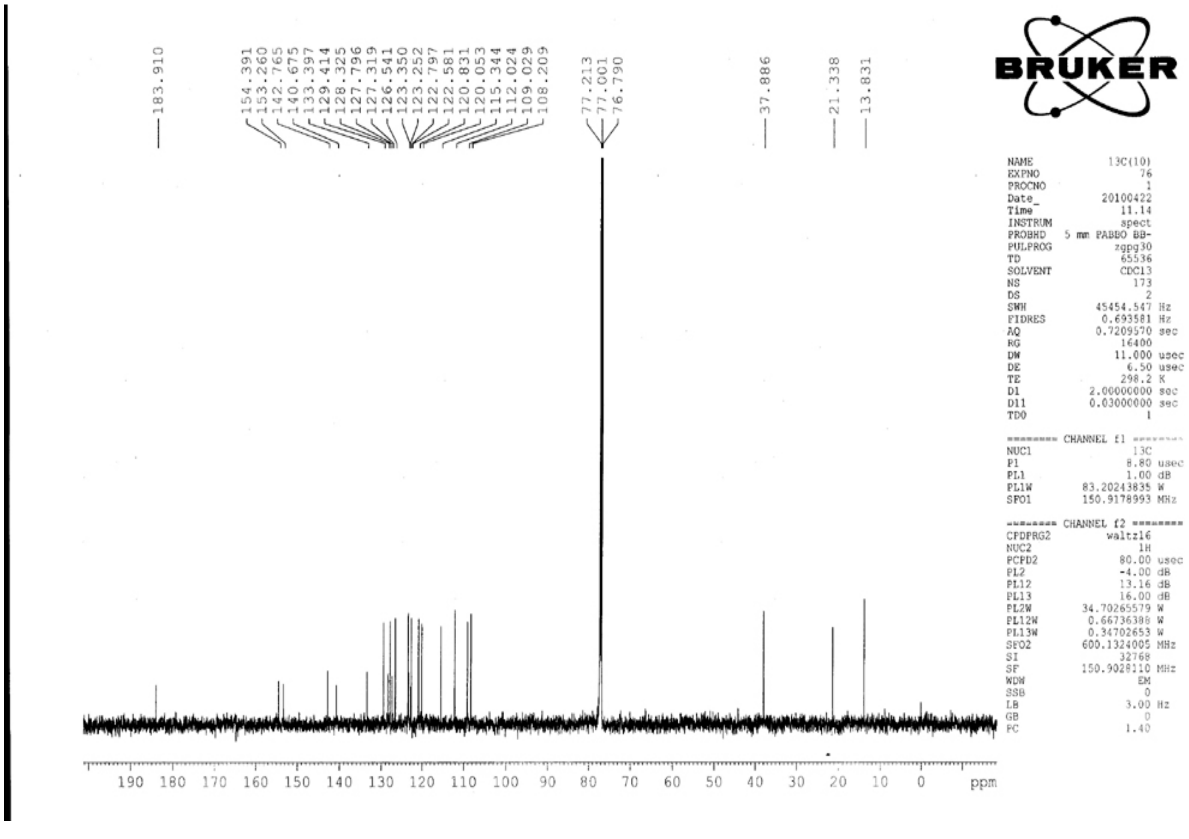

Figure S6: ^{13}C NMR spectrum of **3c**.

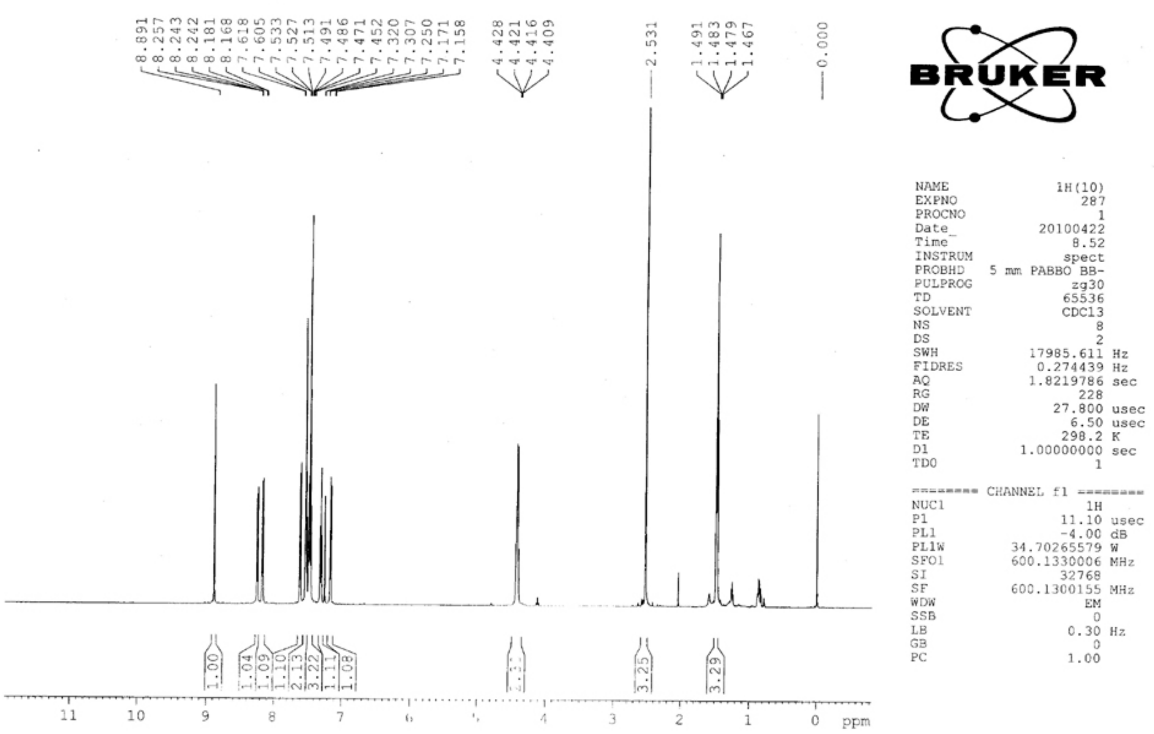

Figure S7: ^{1}H NMR spectrum of **3d**

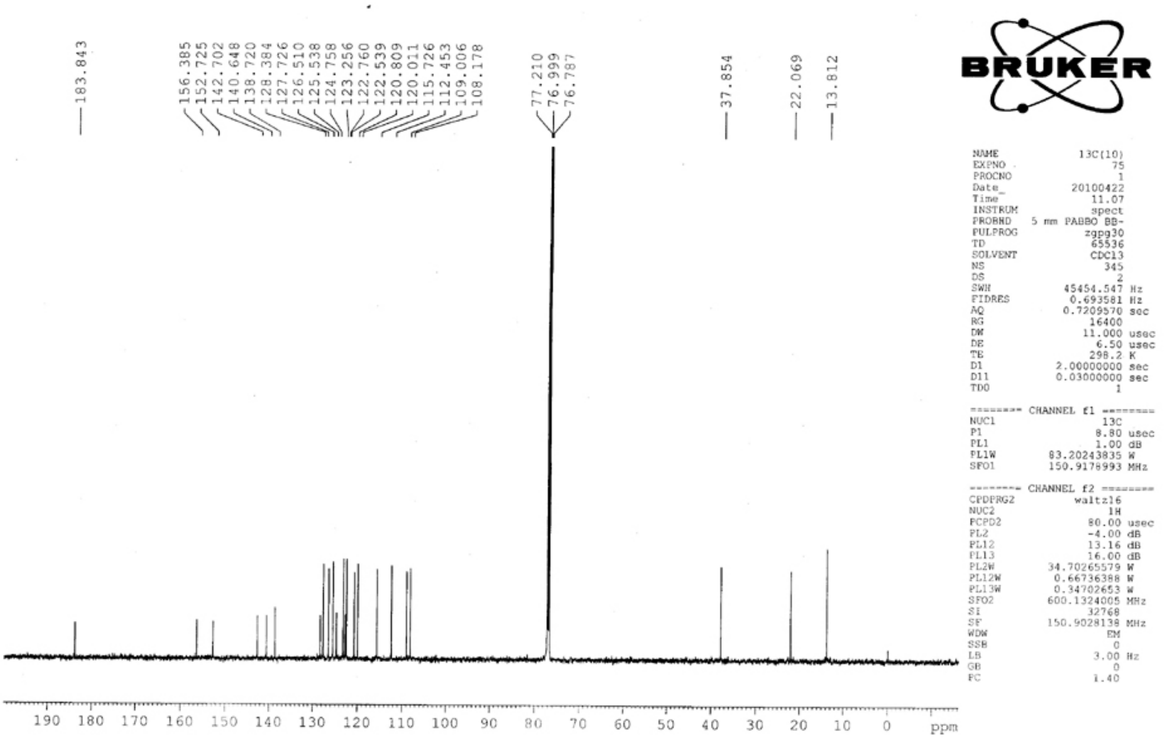

Figure S8: ^{13}C NMR spectrum of **3d**.

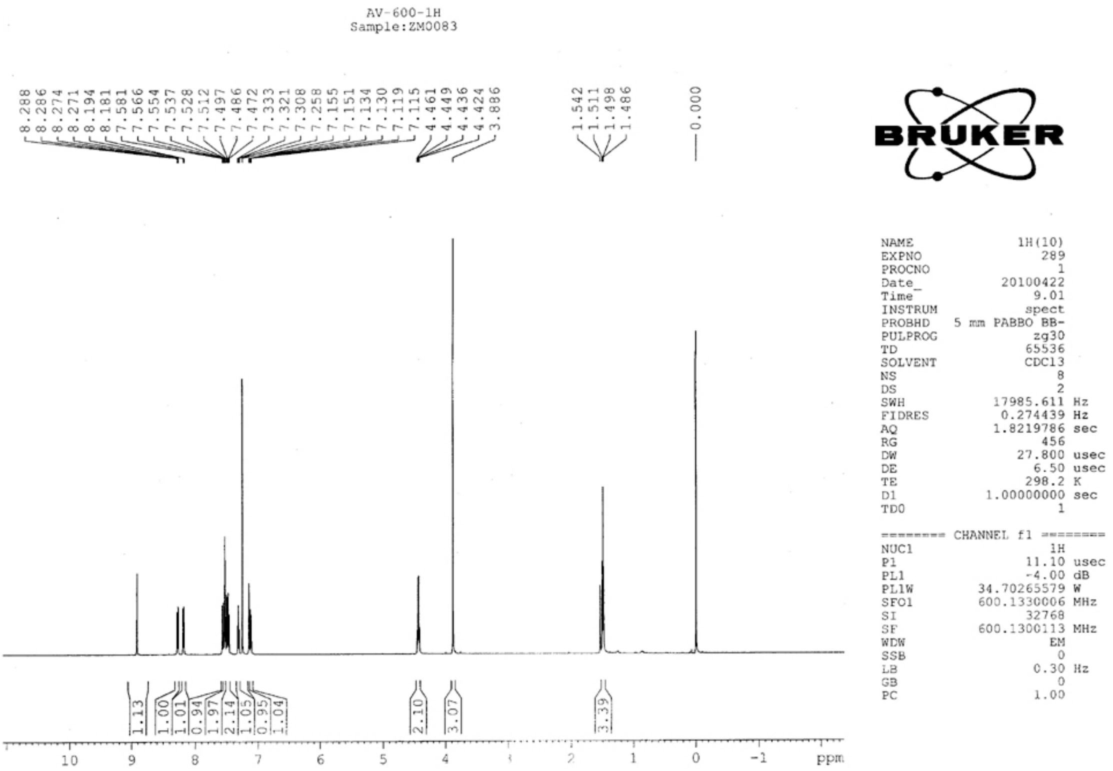

Figure S9: ^{1}H NMR spectrum of **3e**.

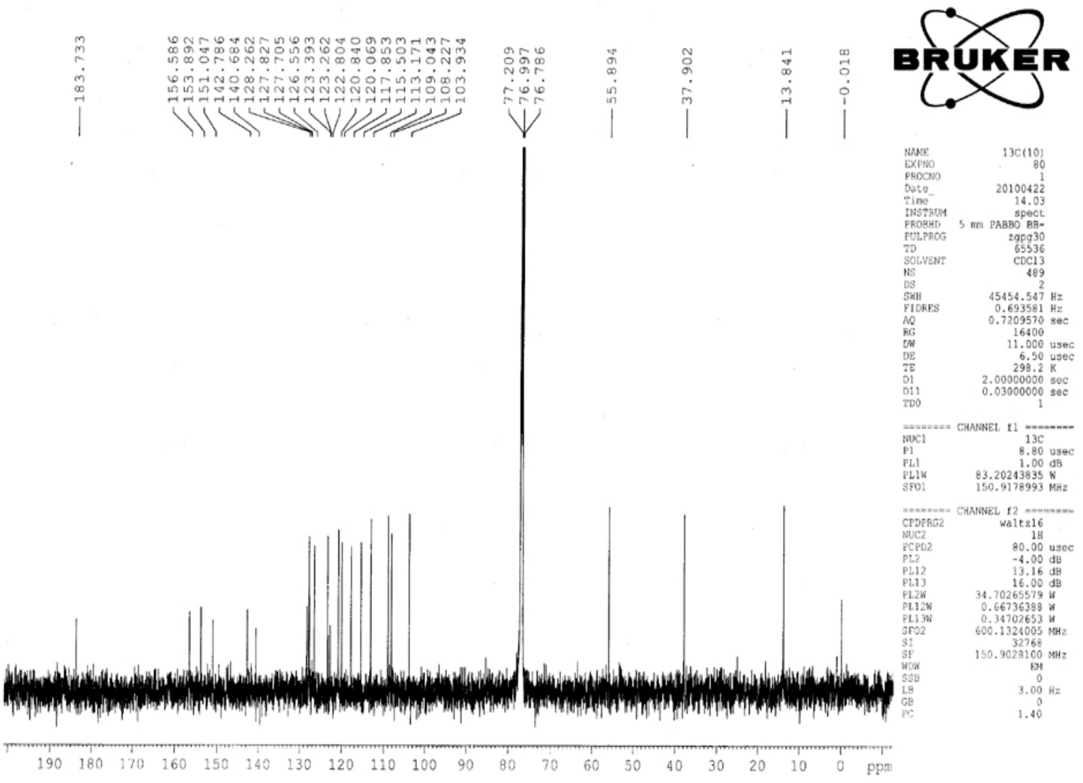

Figure S10: ^{13}C NMR spectrum of **3e**.

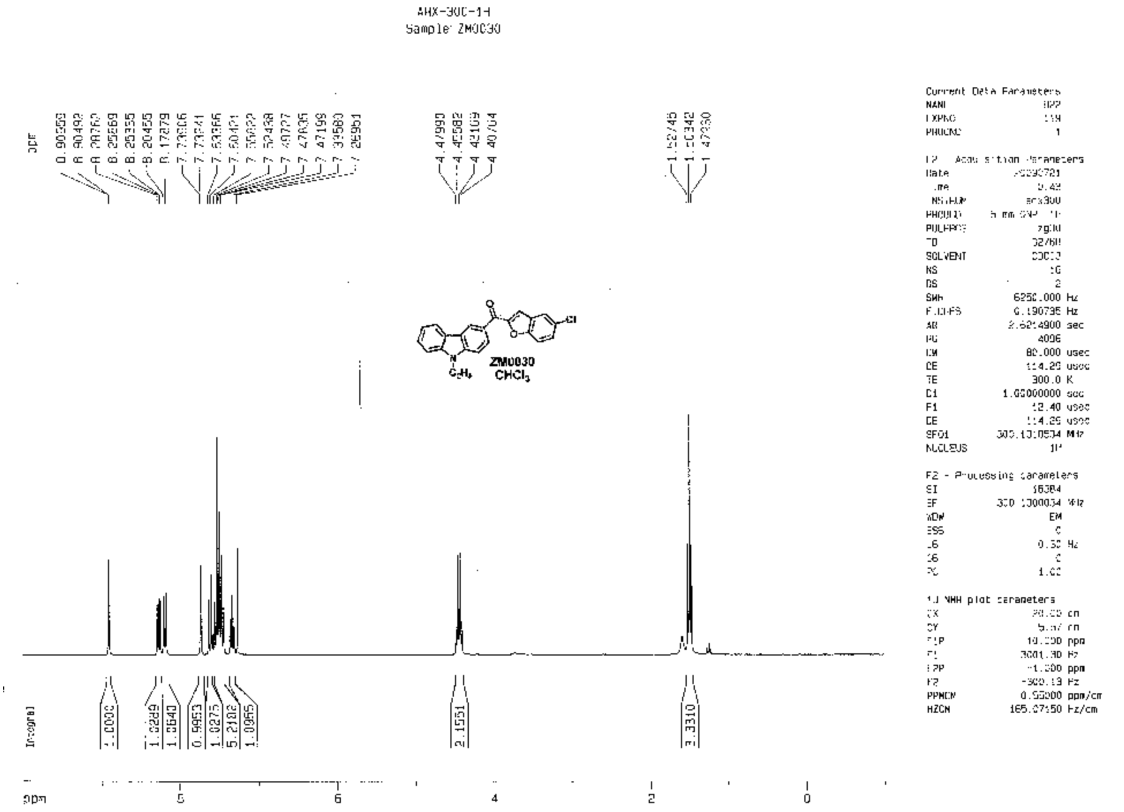

Figure S11: ^{1}H NMR spectrum of **3f**.

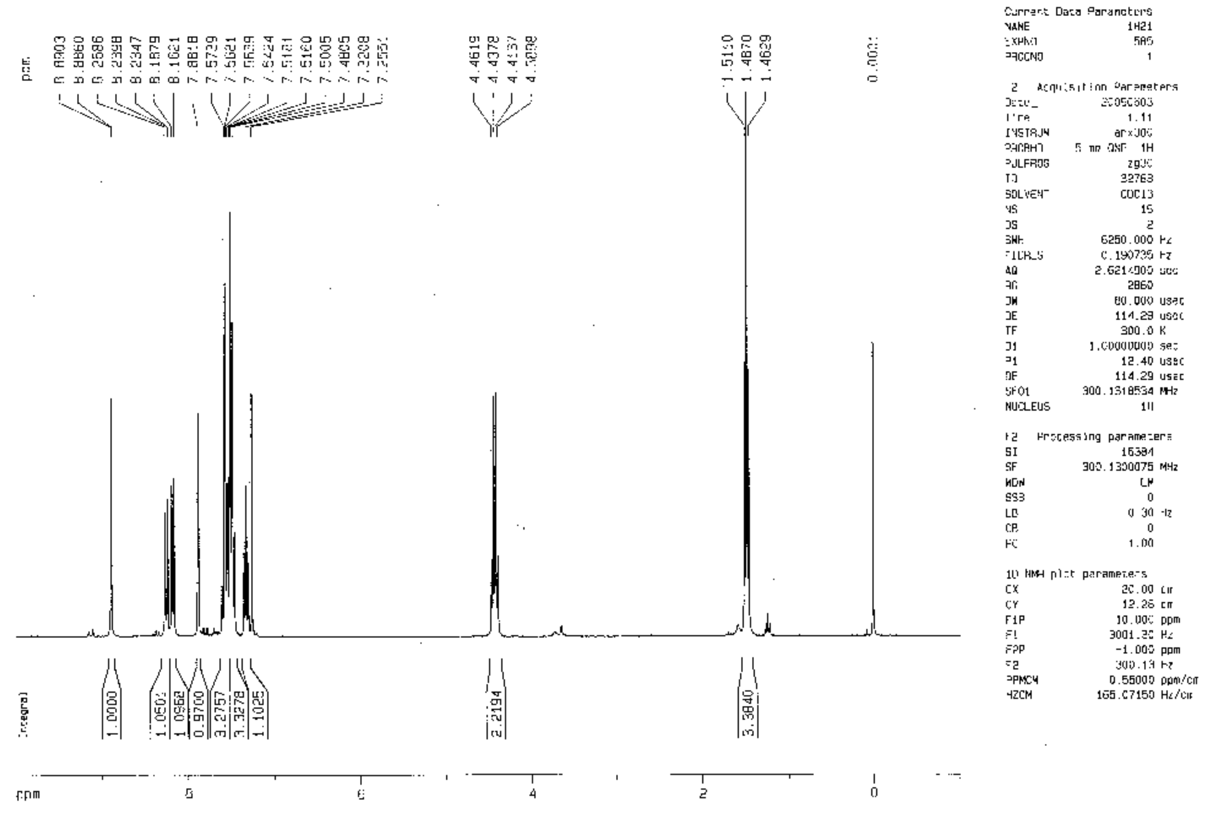

Figure S12: ^{1}H NMR spectrum of **3g**.

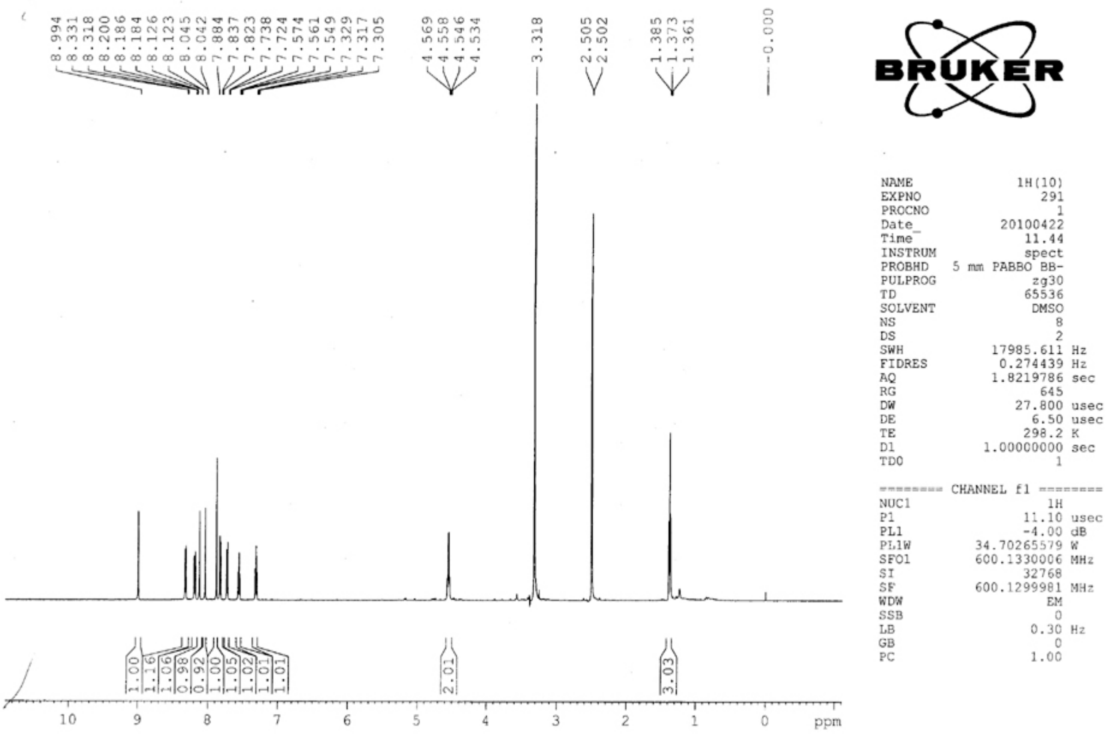

Figure S13: ^{13}C NMR spectrum of **3h**.

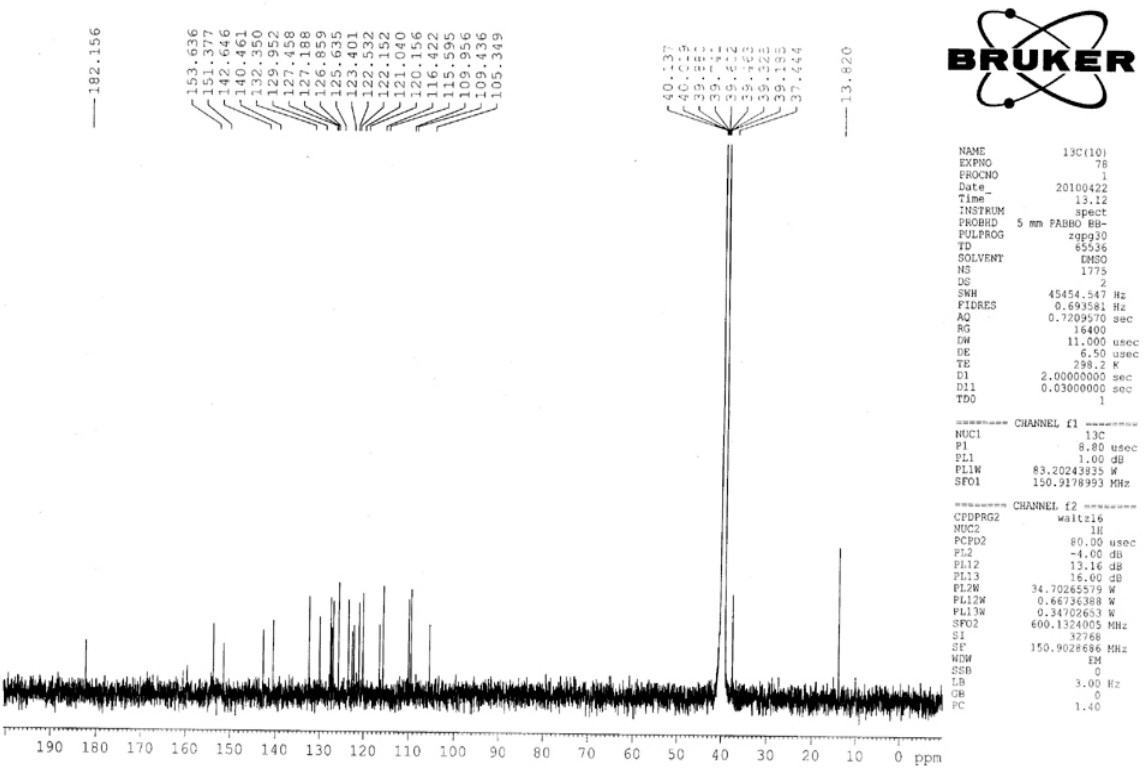

Figure S14: ^{13}C NMR spectrum of **3h**.

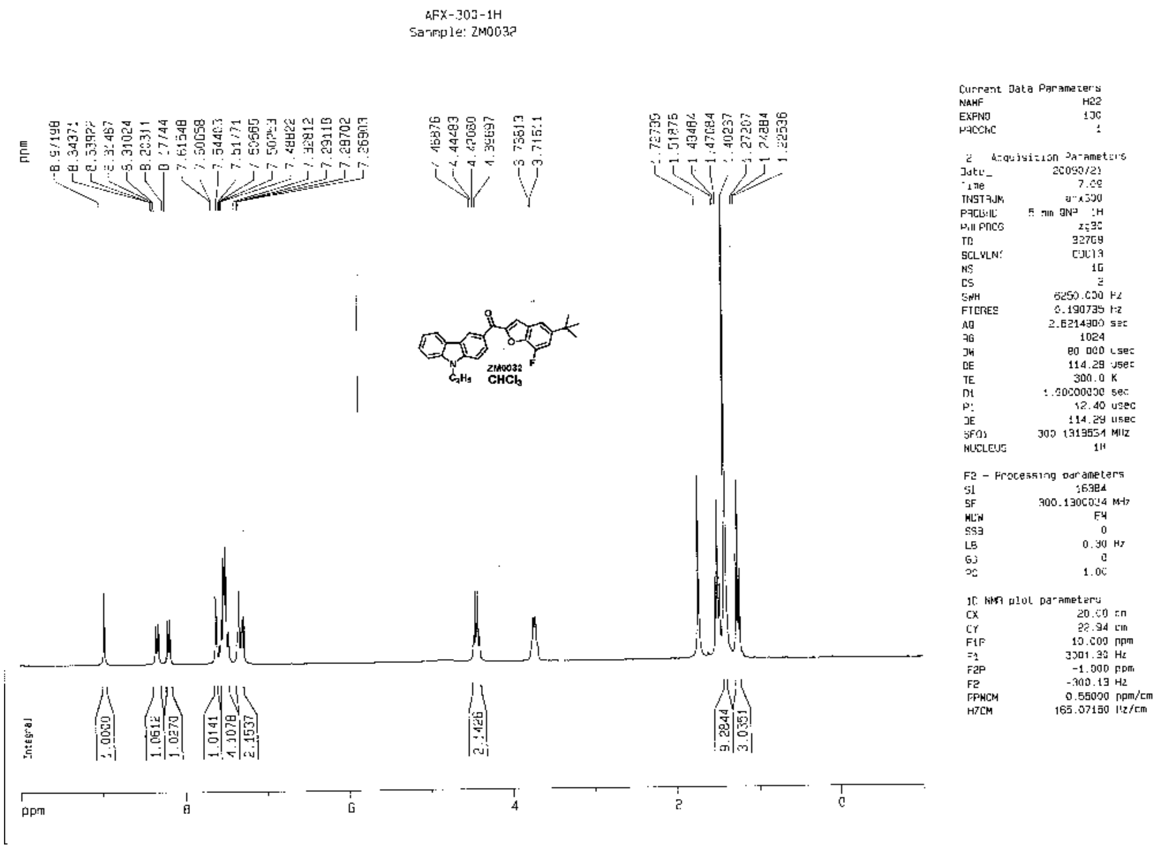

Figure S15: ^{1}H NMR spectrum of **3i**.

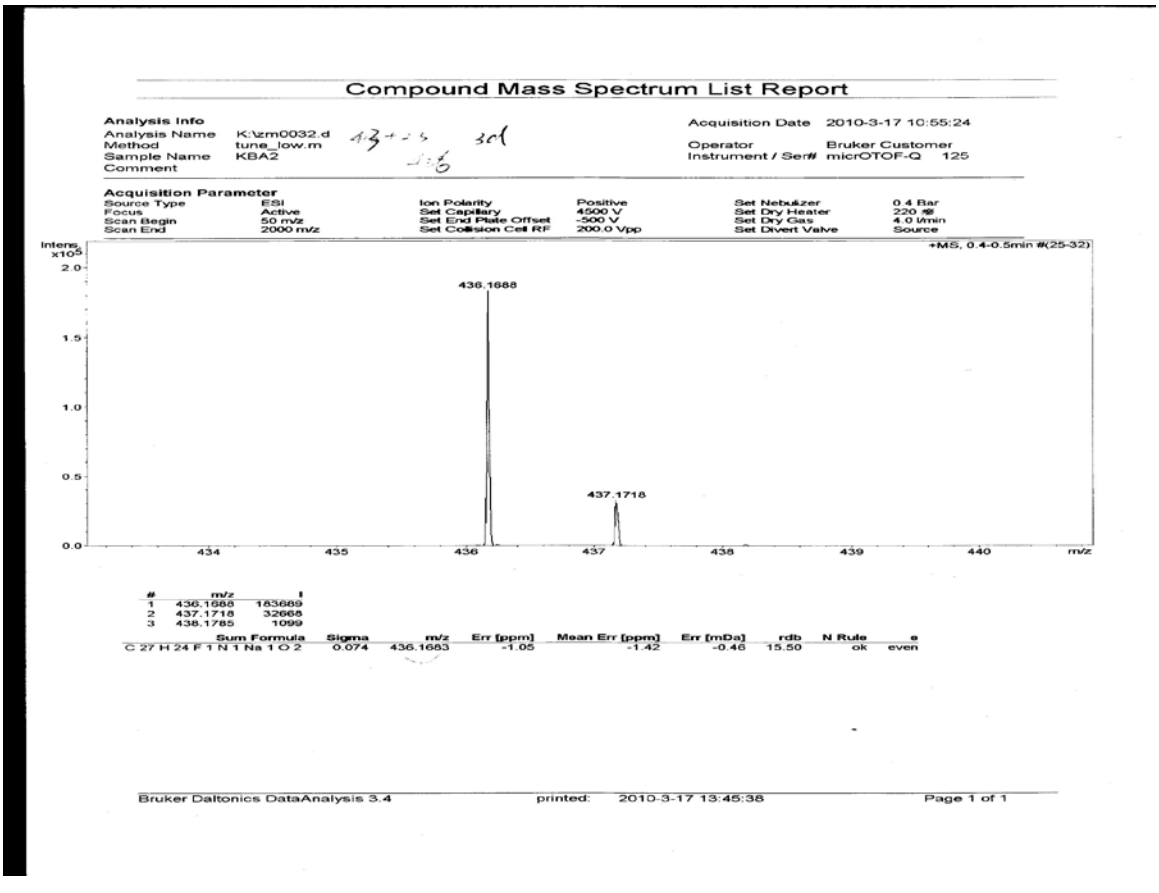

Figure S16: HRMS spectrum of **3i**.

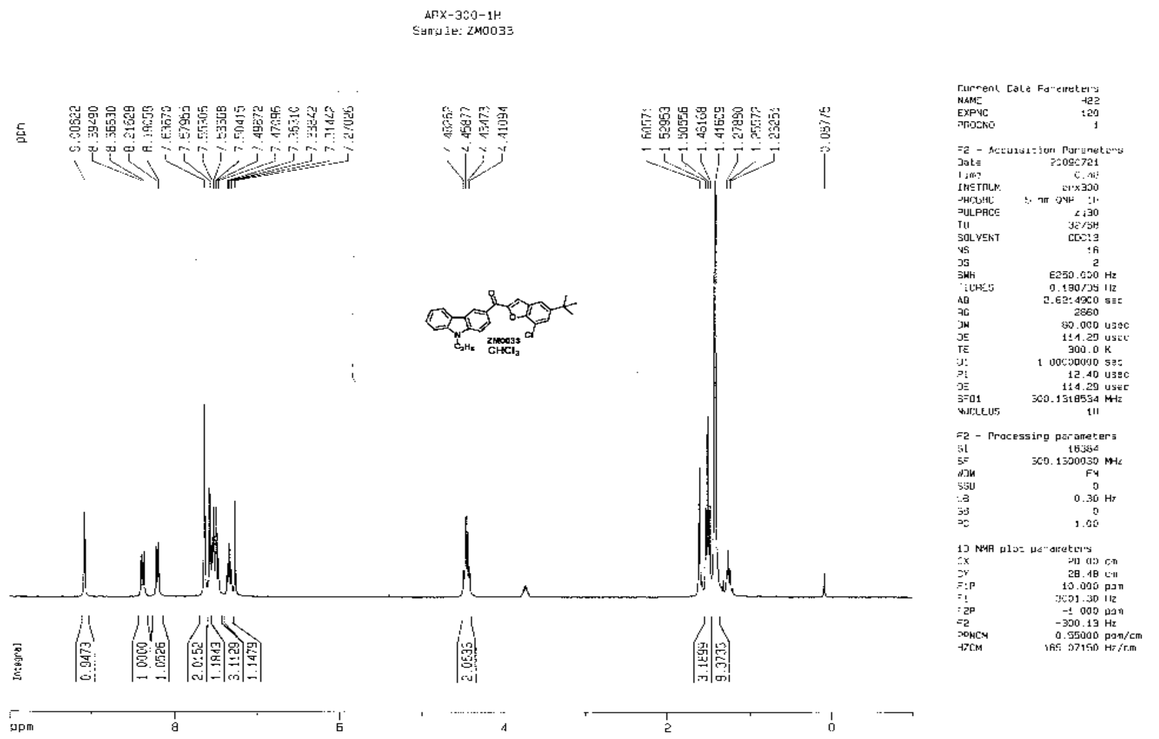

Figure S17: ^{1}H NMR spectrum of **3j**.

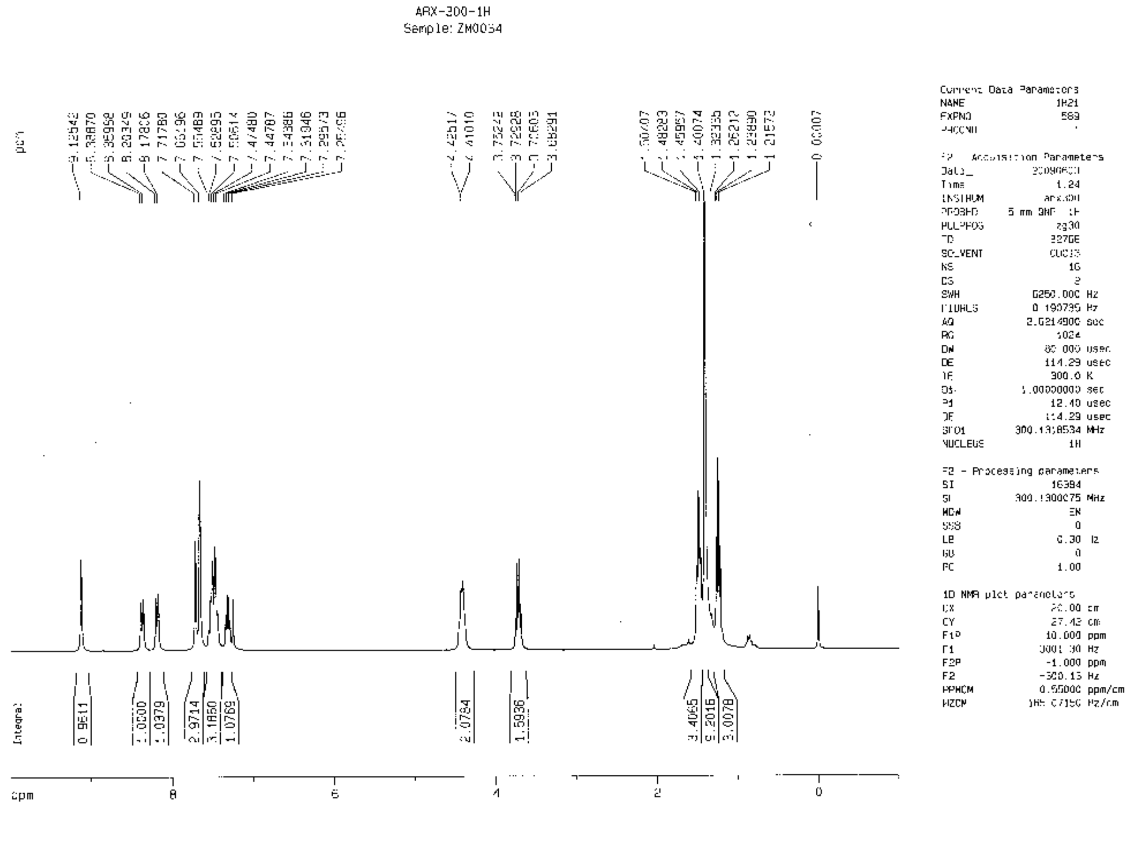

Figure S18: ^{1}H NMR spectrum of **3k**.

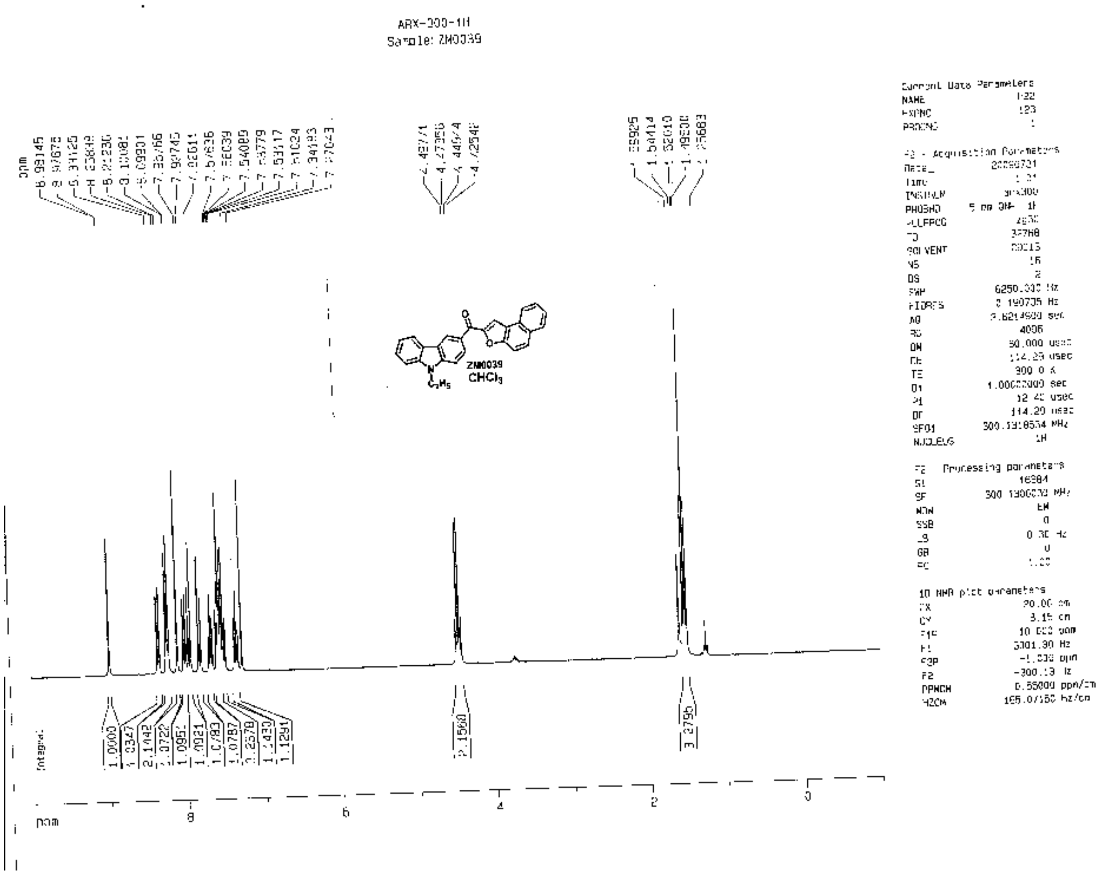

Figure S19: ^{1}H NMR spectrum of **3l**.

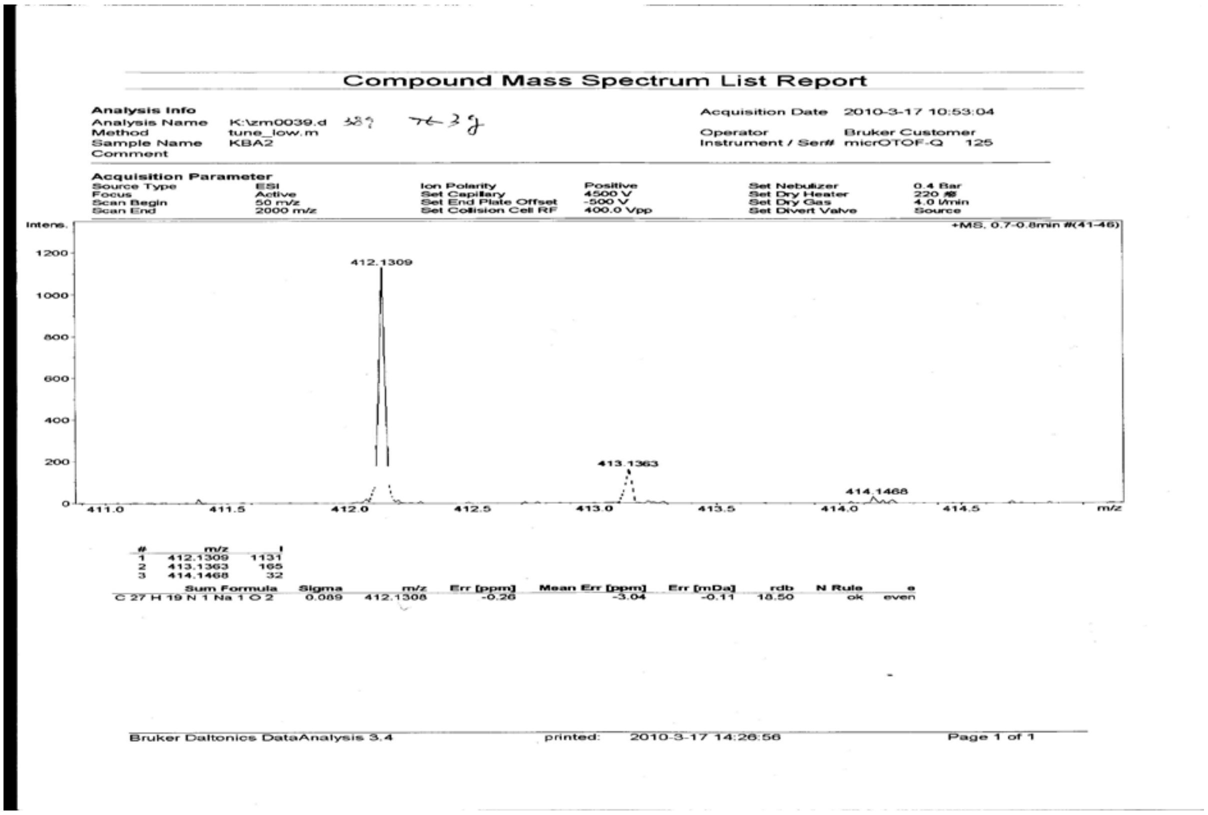

Figure S20: HRMS spectrum of **3l**.

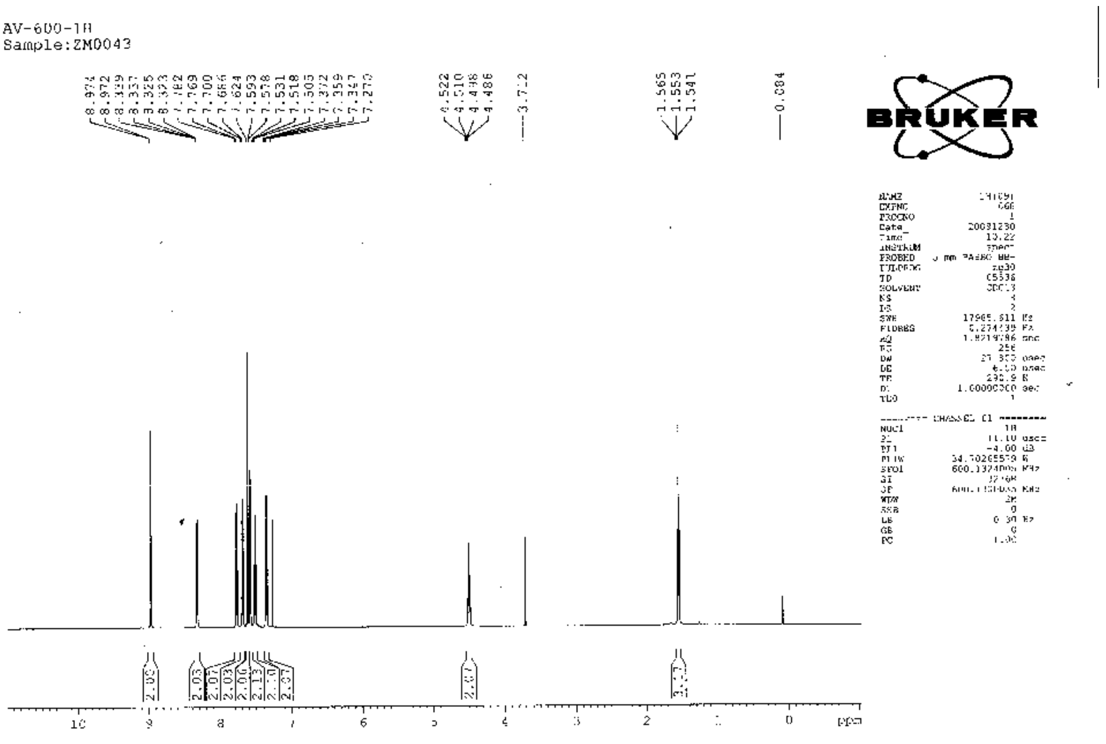

Figure S21: ^{1}H NMR spectrum of **5a**.

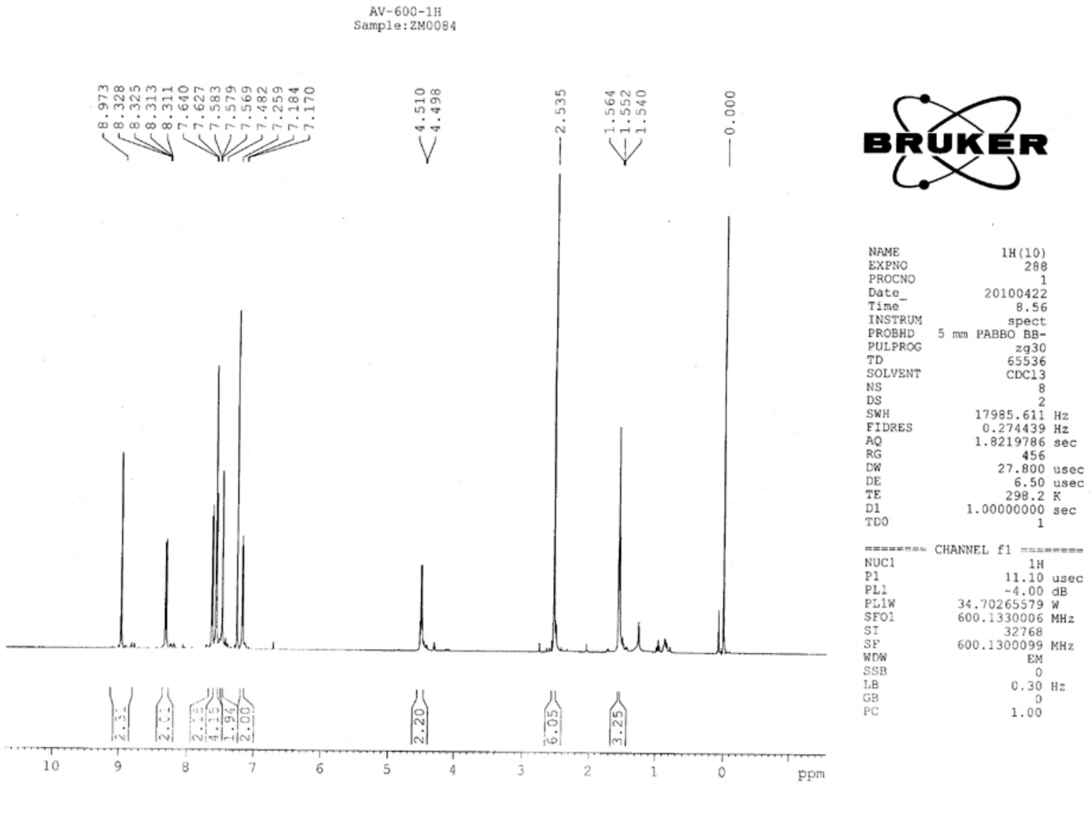

Figure S22: ^{1}H NMR spectrum of **5b**.

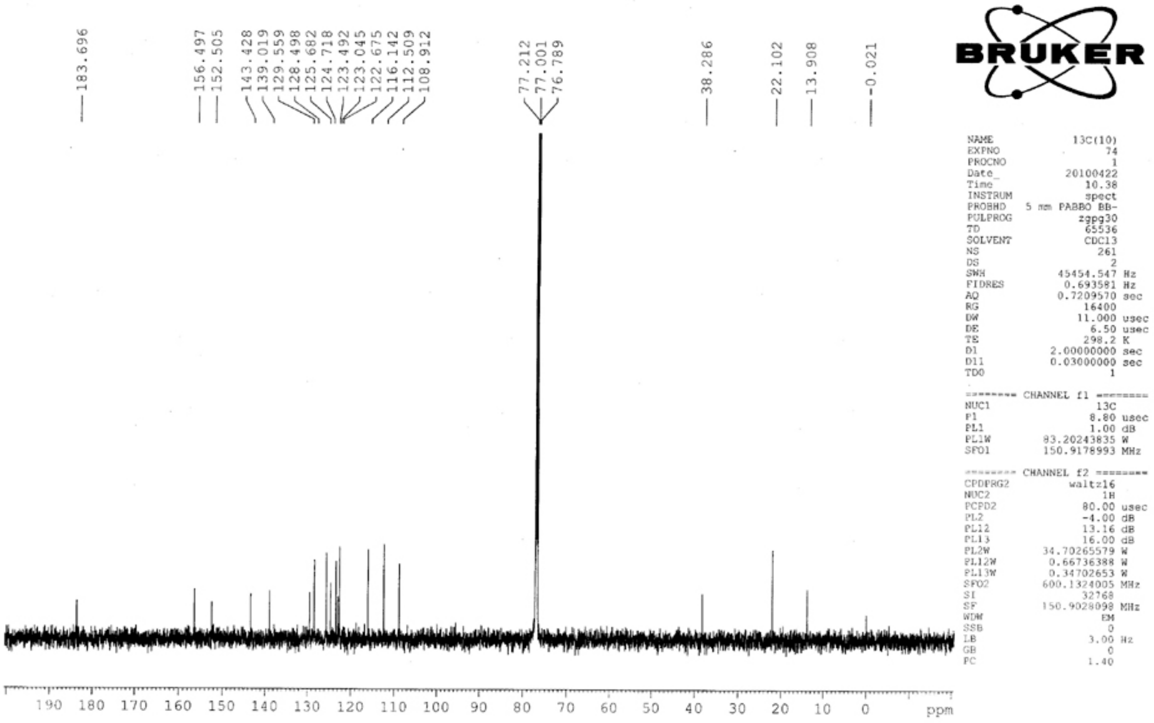

Figure S23: ^{13}C NMR spectrum of **5b**.

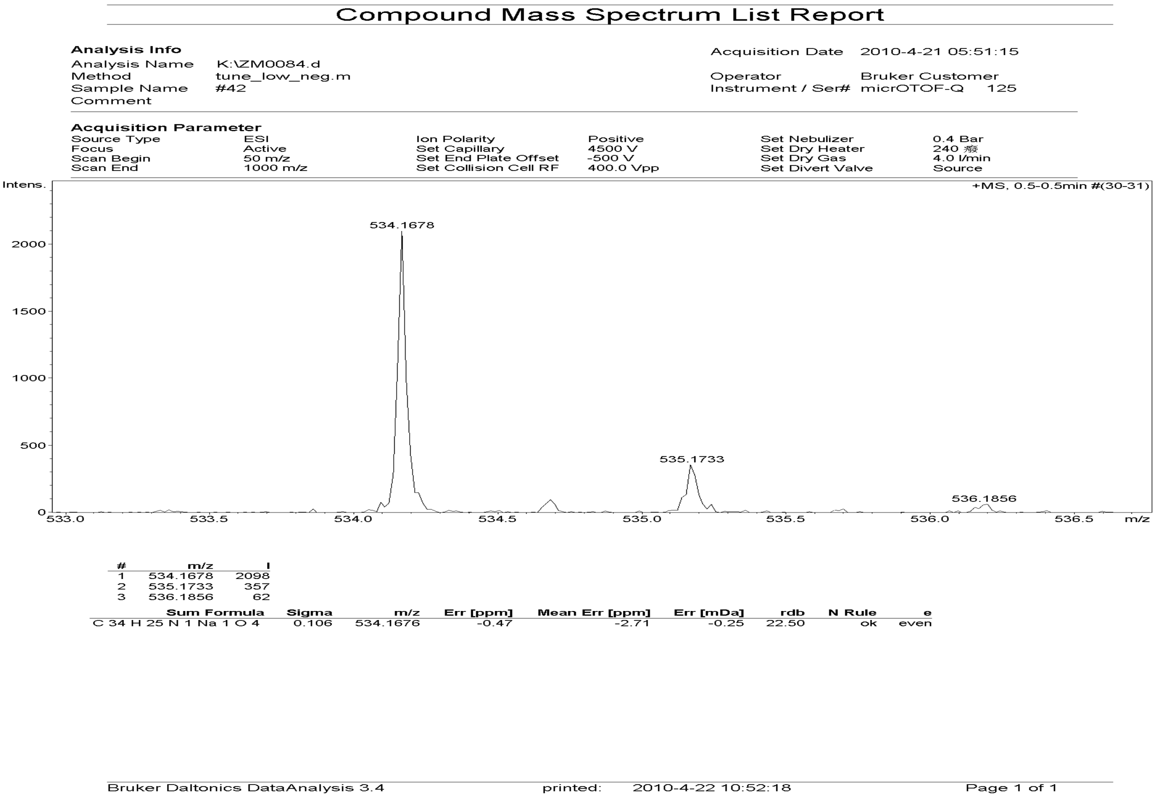

Figure S24: HRMS spectrum of **5b**.

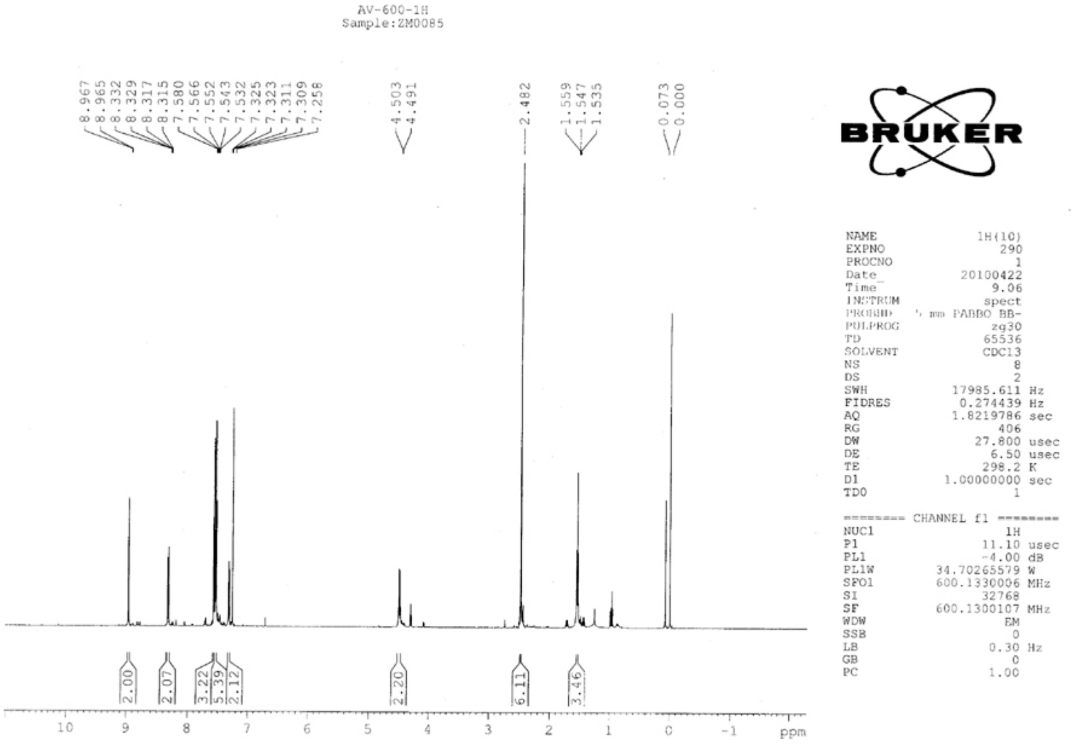

Figure S25: ^{1}H NMR spectrum of **5c**.

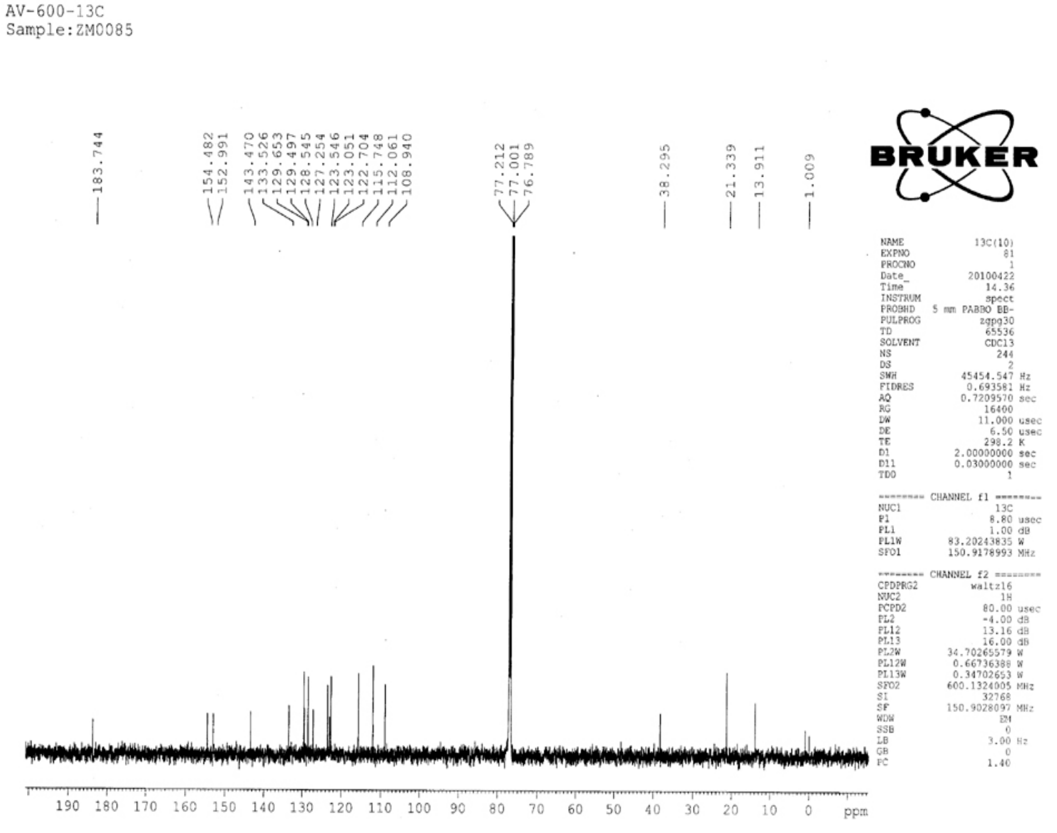

Figure S26: ^{13}C NMR spectrum of **5c**.

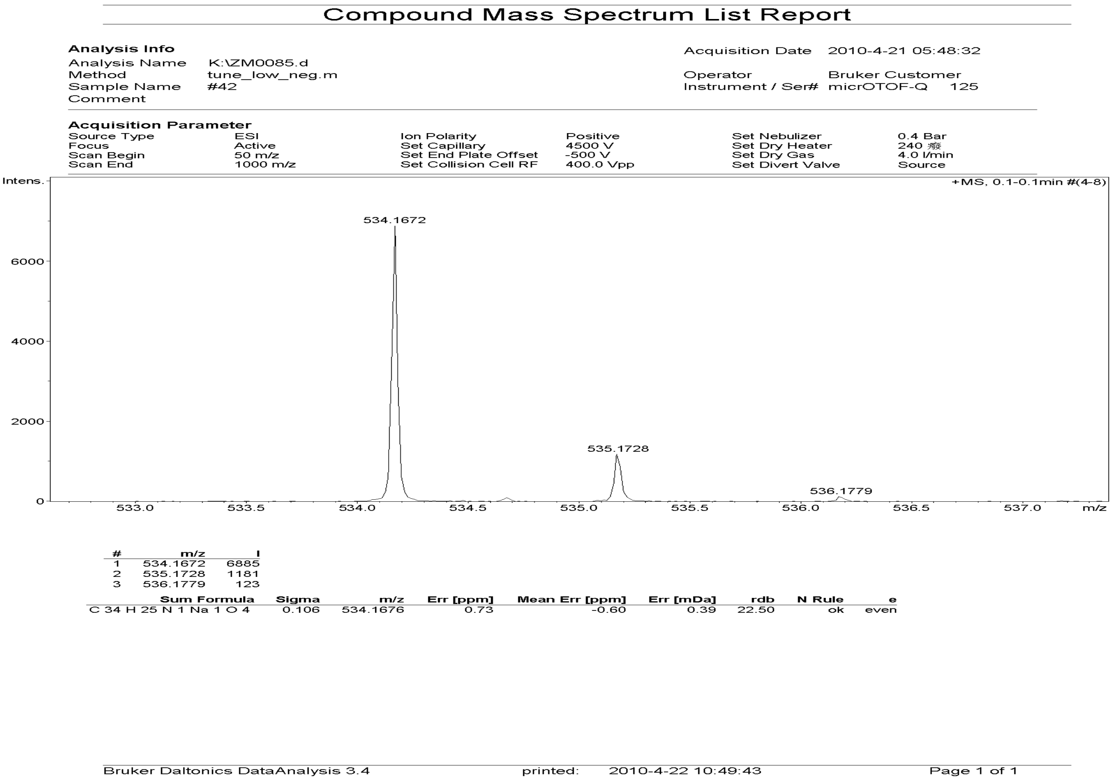

Figure S27: HRMS spectrum of **5c**.

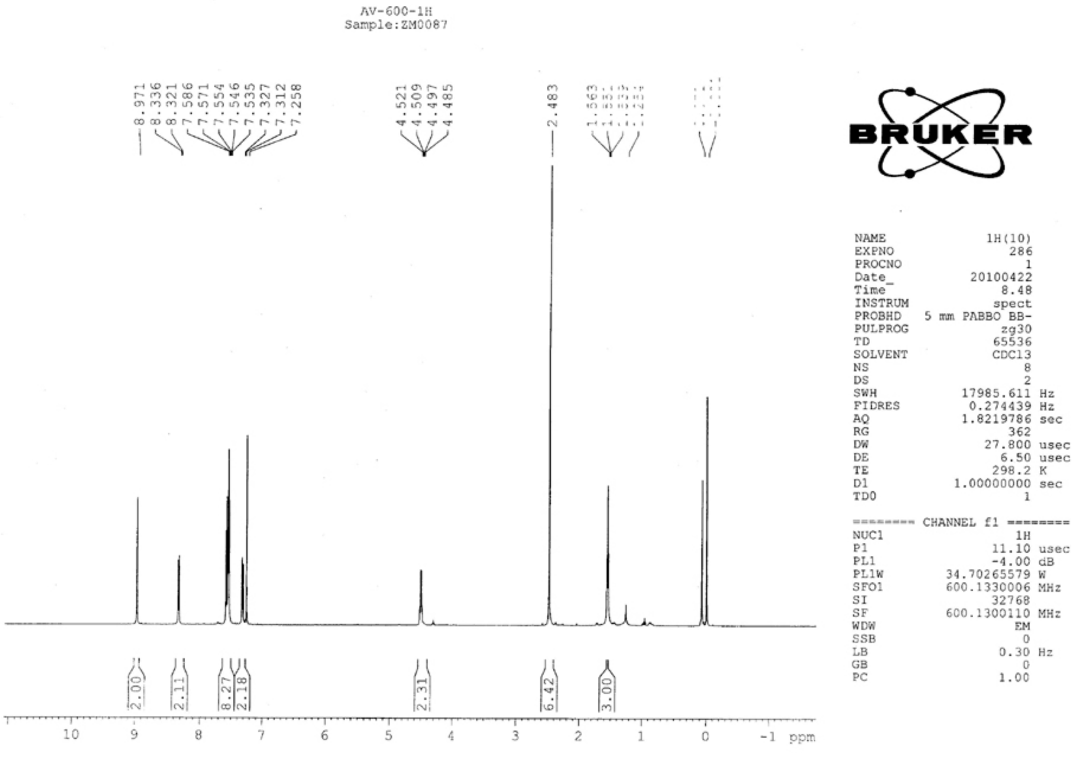

Figure S28: ^{1}H NMR spectrum of **5d**.

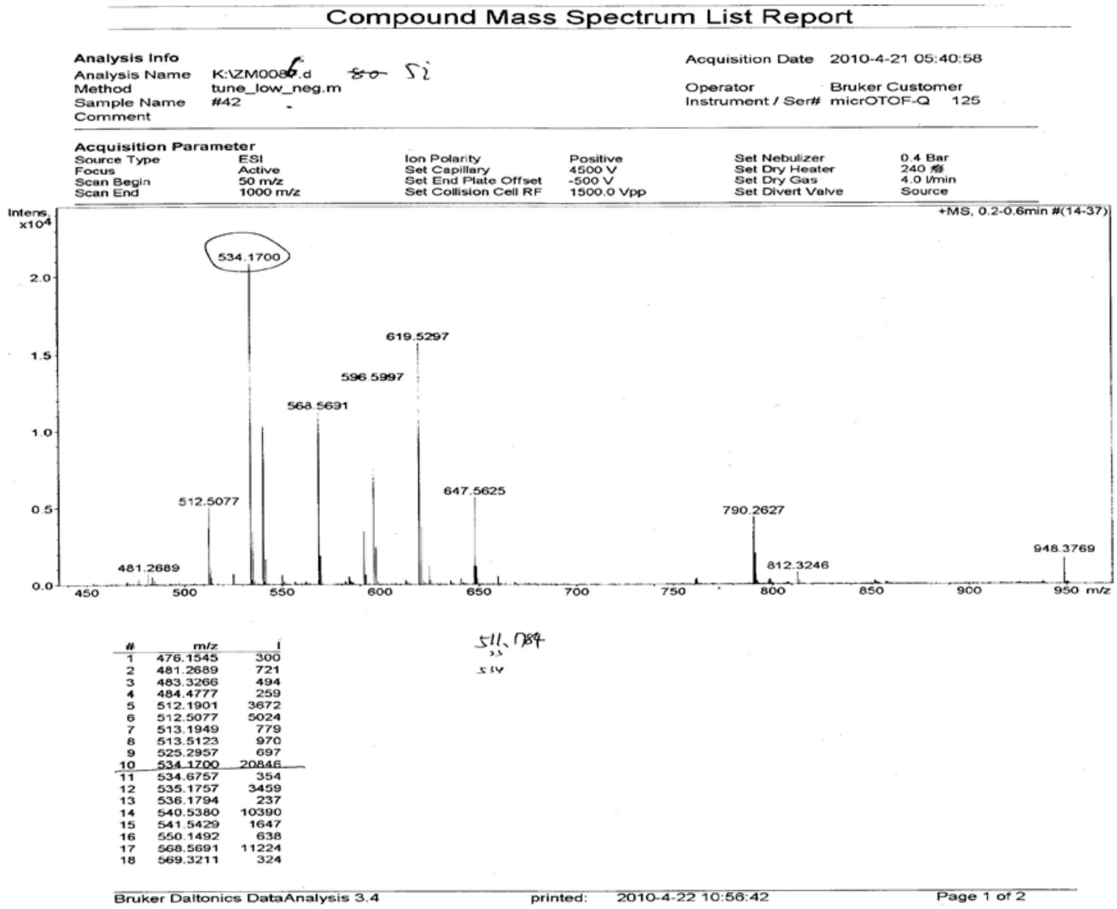

Figure S29: HRMS spectrum of **5d**.

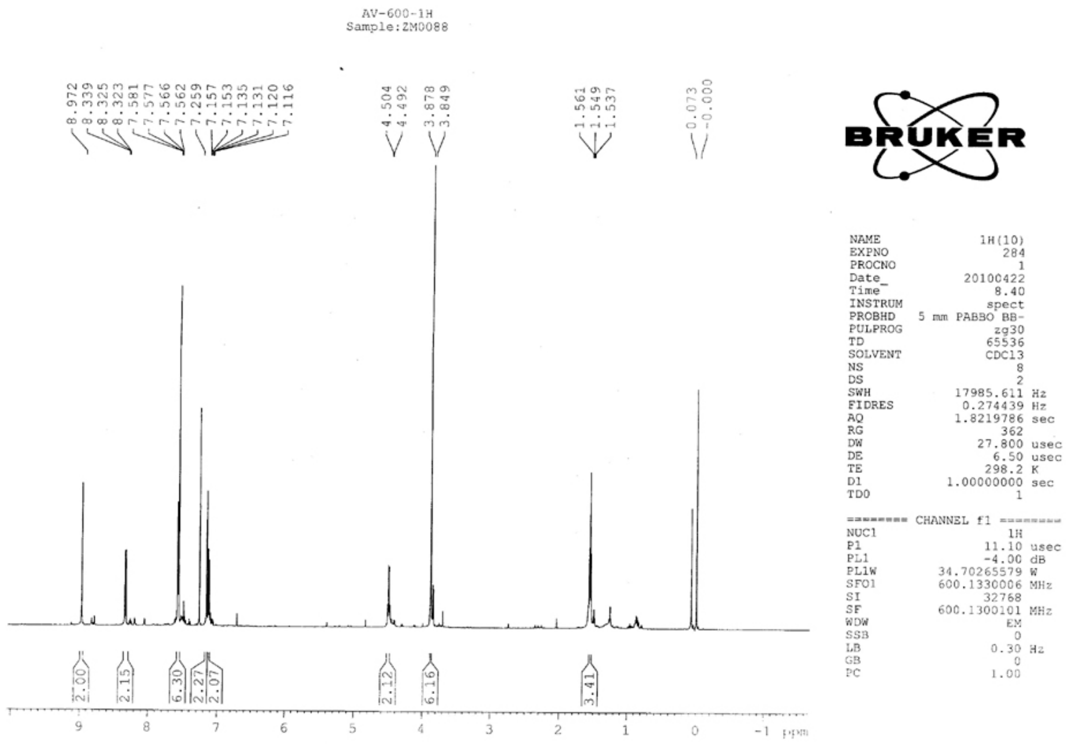

Figure S30: ^{1}H NMR spectrum of **5e**.

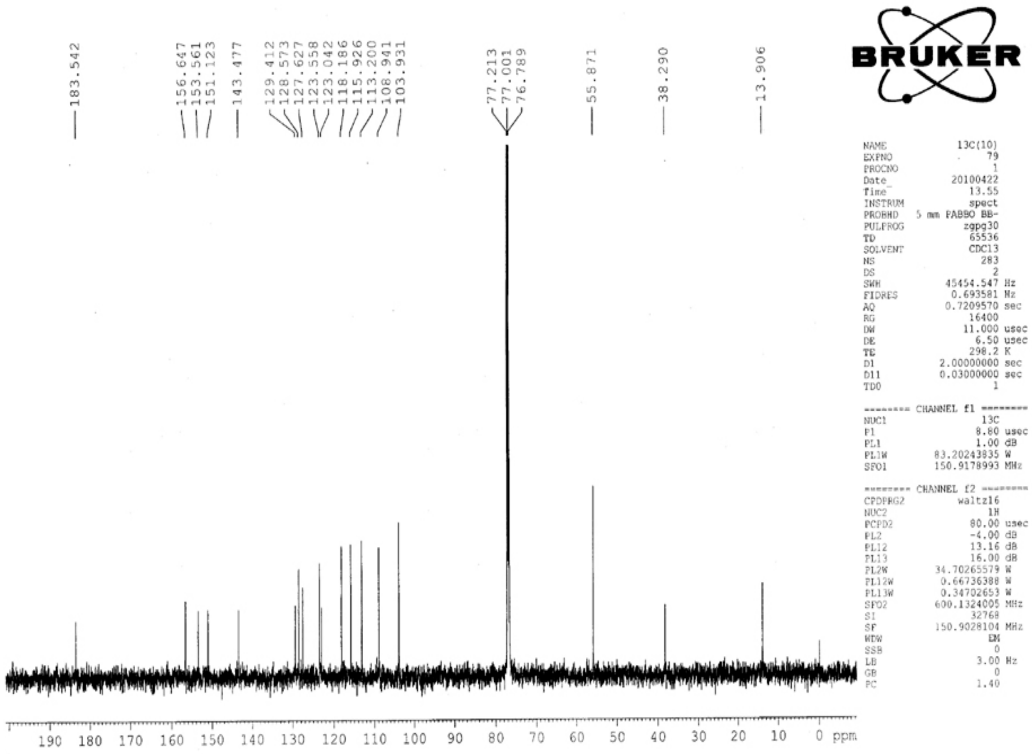

Figure S31: ^{13}C NMR spectrum of **5e**.

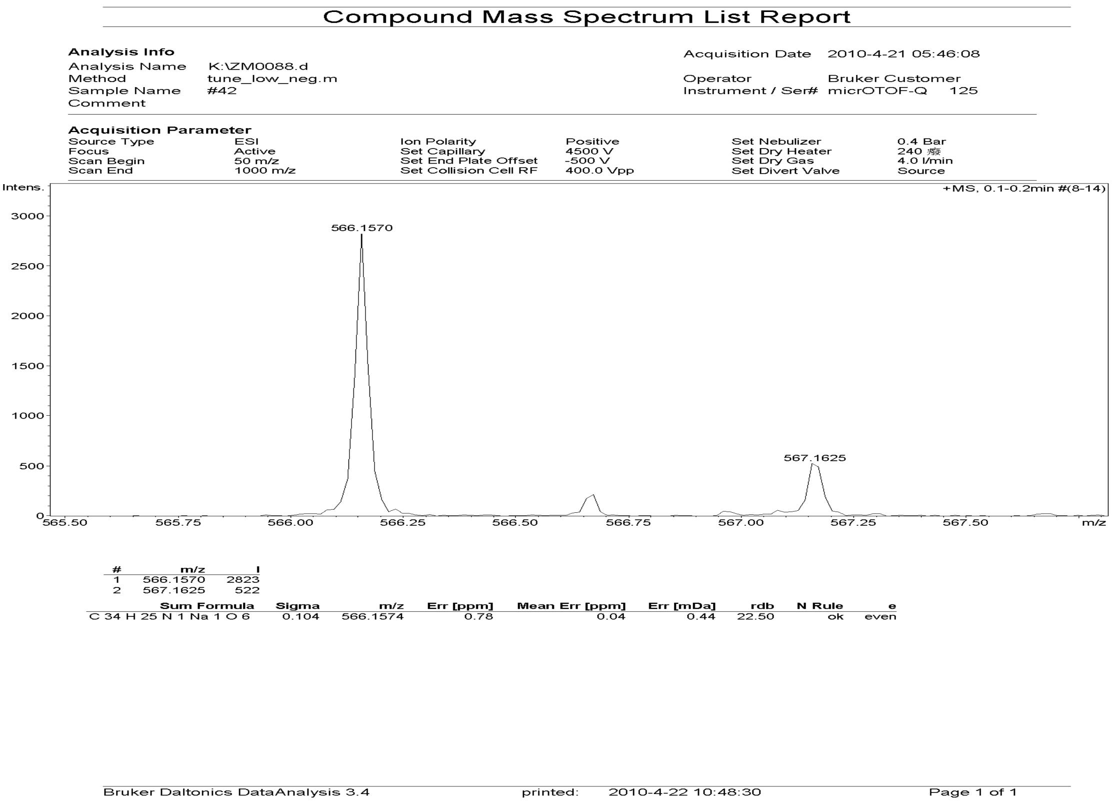

Figure S32: HRMS spectrum of **5e**.

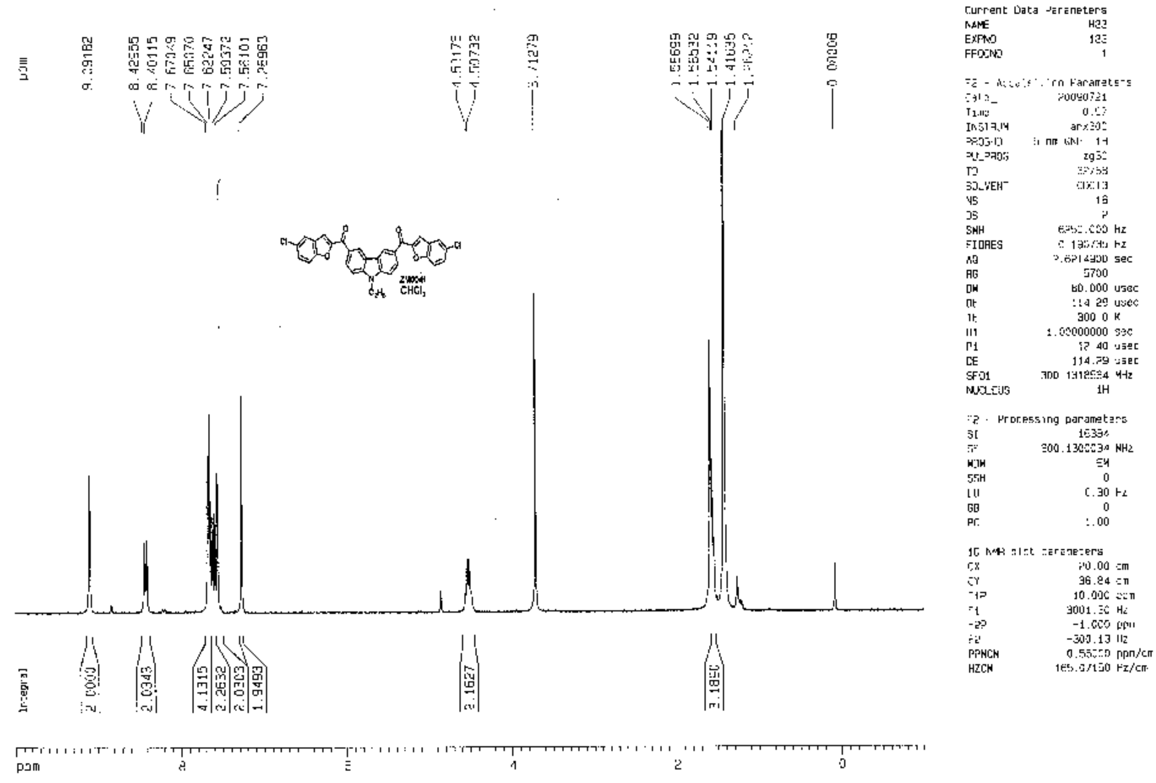

Figure S33: ^{1}H NMR spectrum of **5f.**

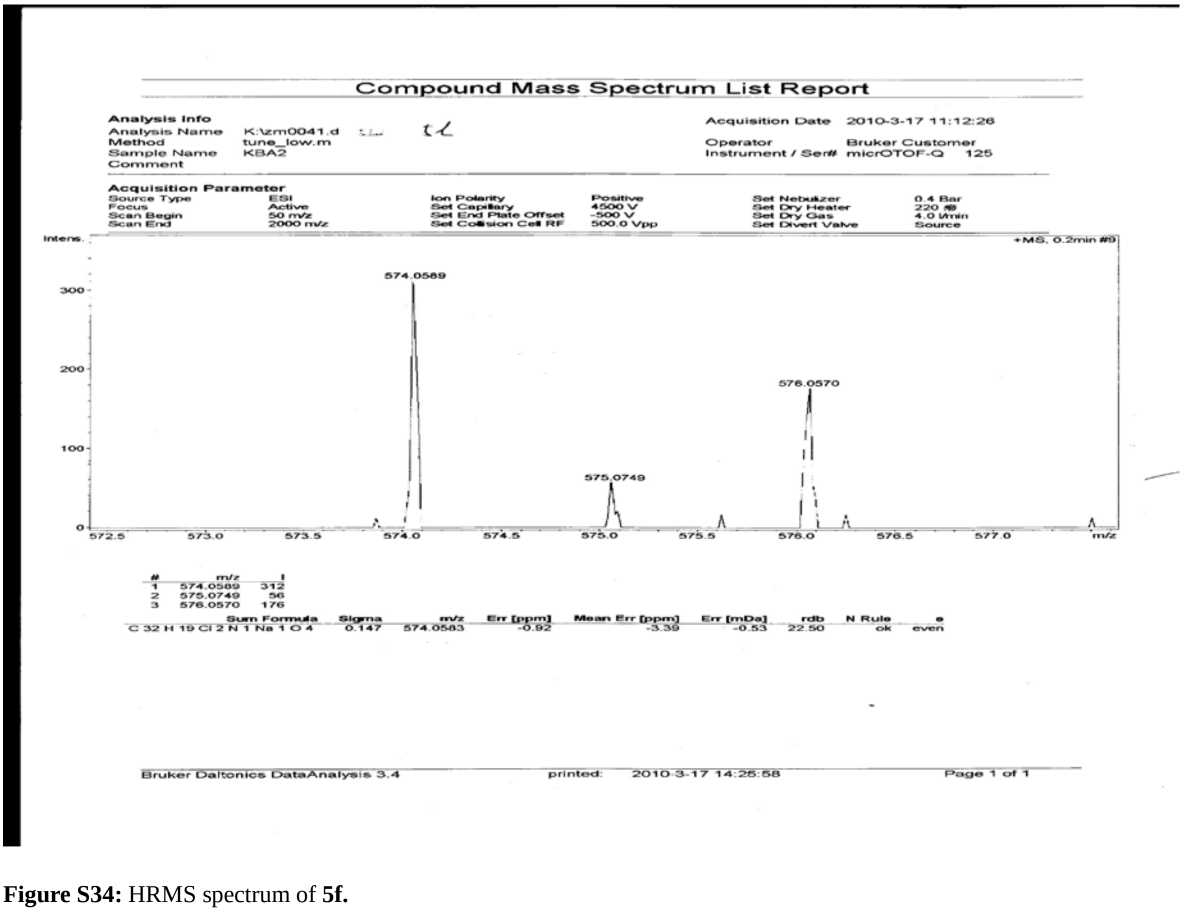

Figure S34: HRMS spectrum of **5f.**

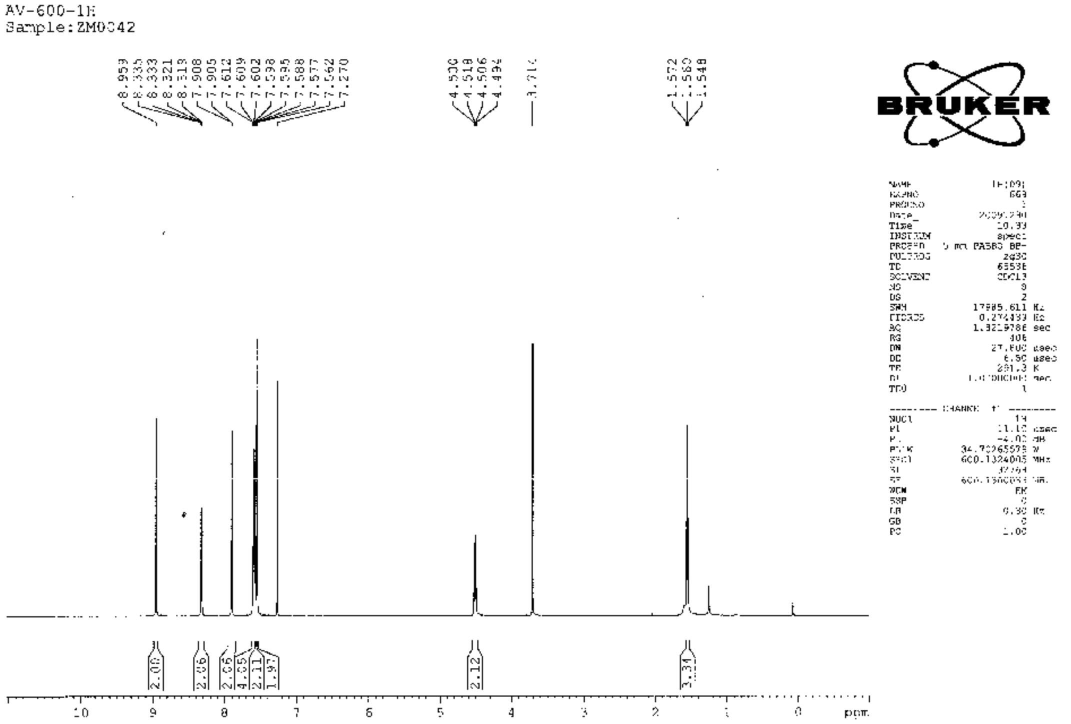

Figure S35: ^{1}H NMR spectrum of **5g.**

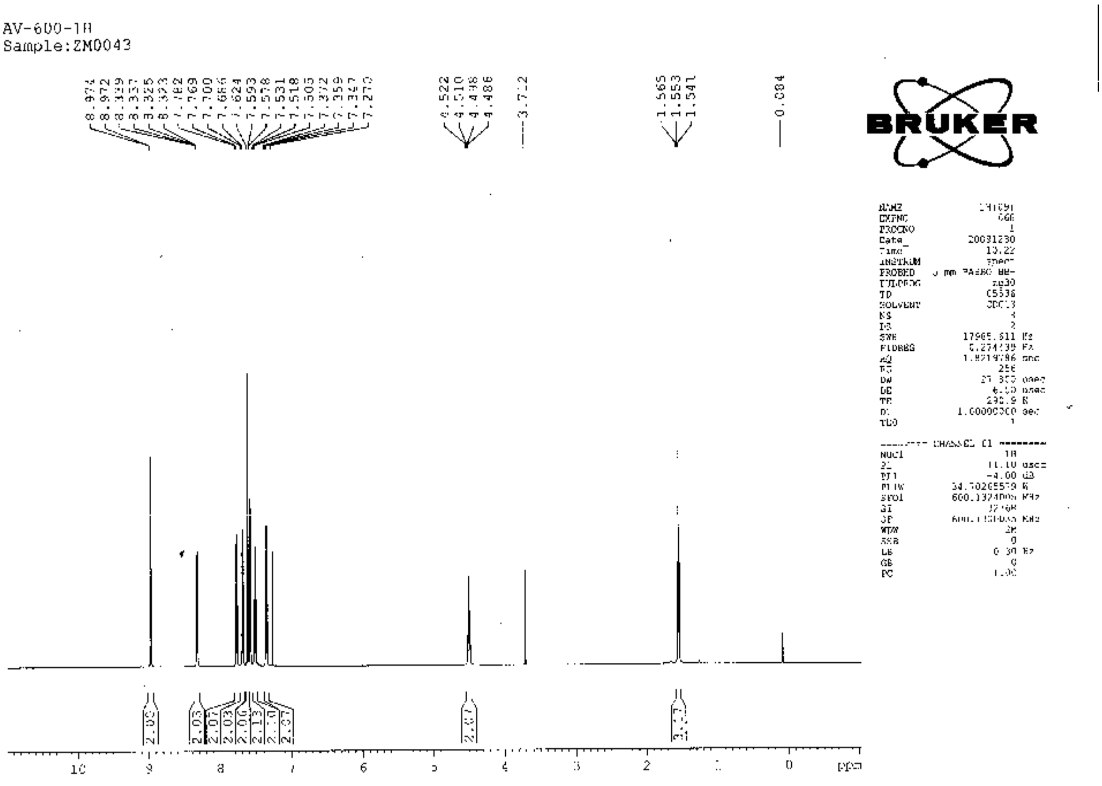

Figure S36: ^{1}H NMR spectrum of **5h.**

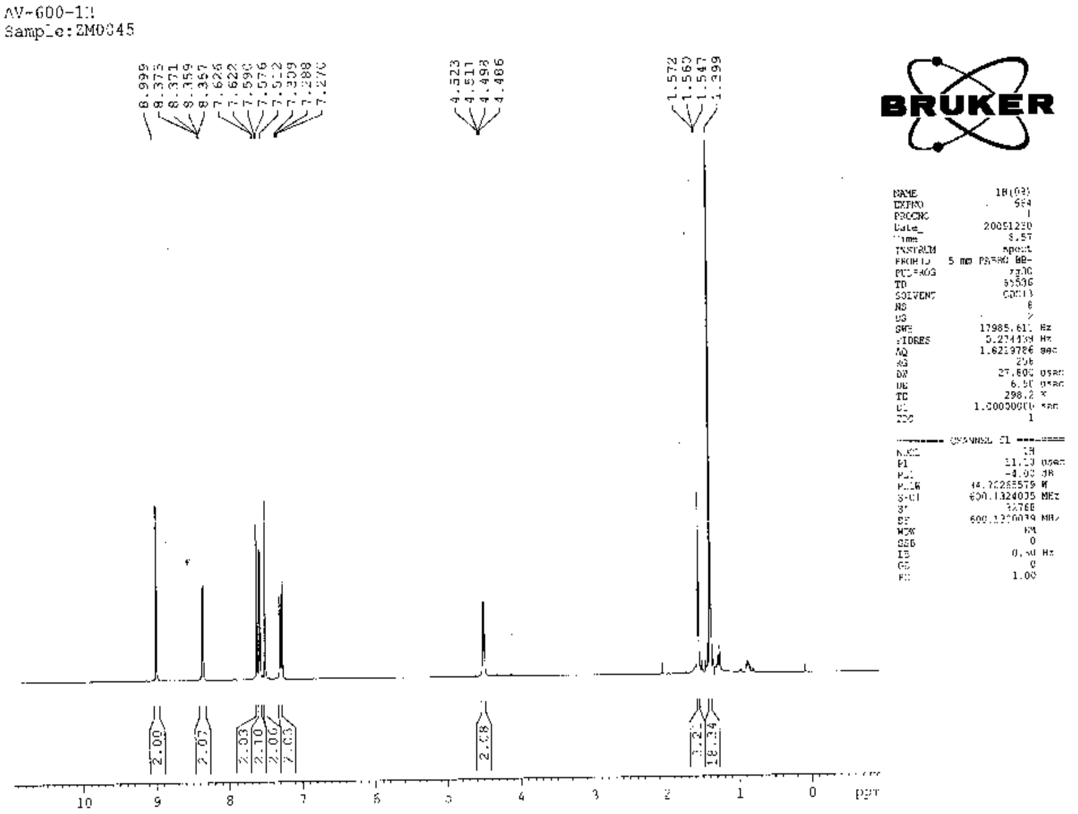

Figure S37: ^{1}H NMR spectrum of **5i**.

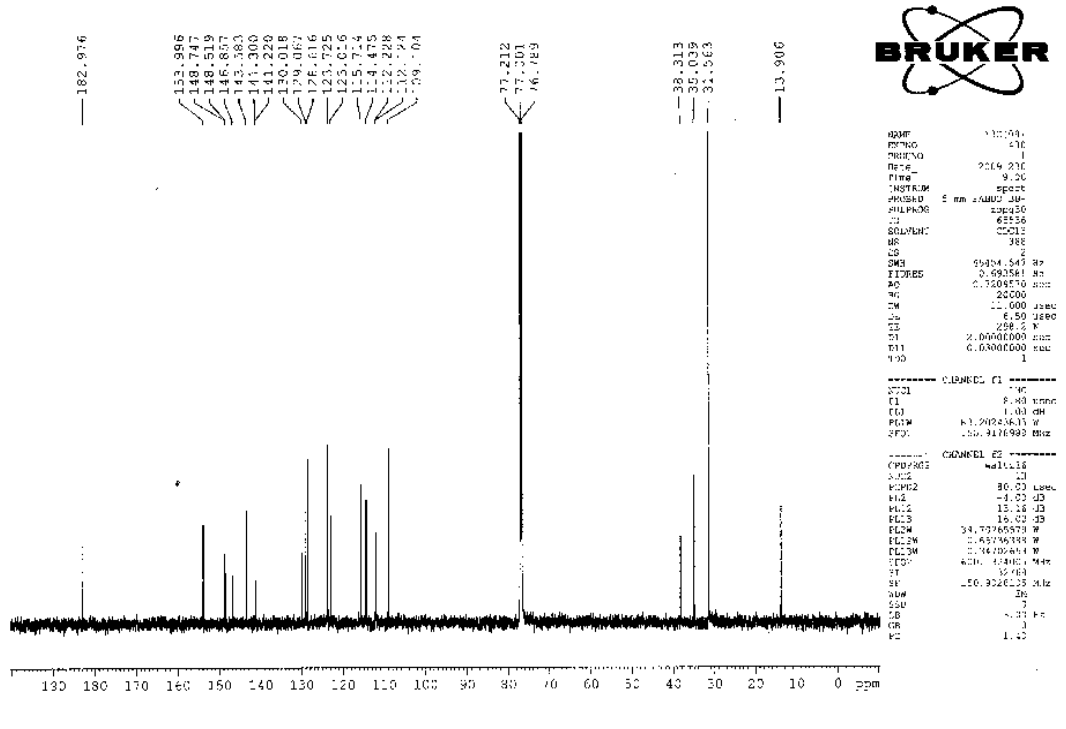

Figure S38: ^{13}C NMR spectrum of **5i**.

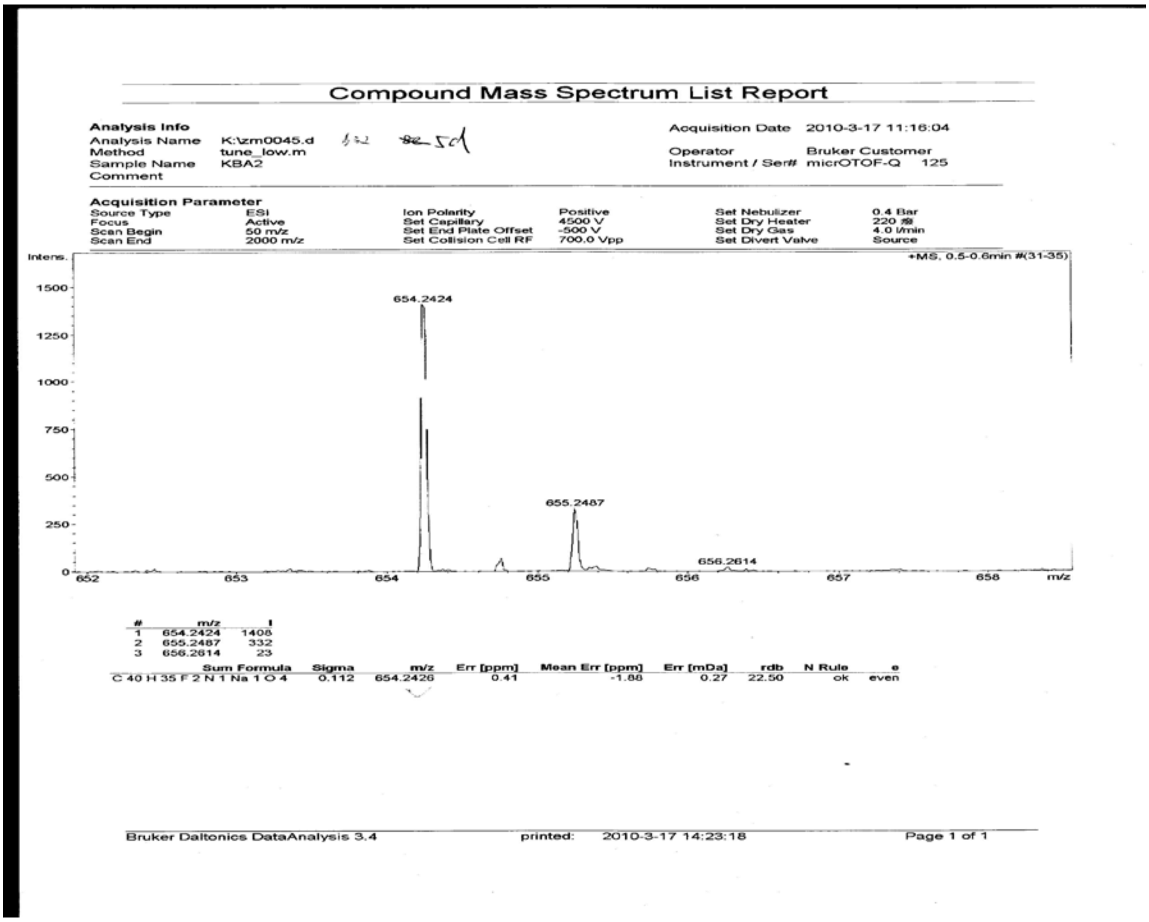

Figure S39: HRMS spectrum of **5i**.

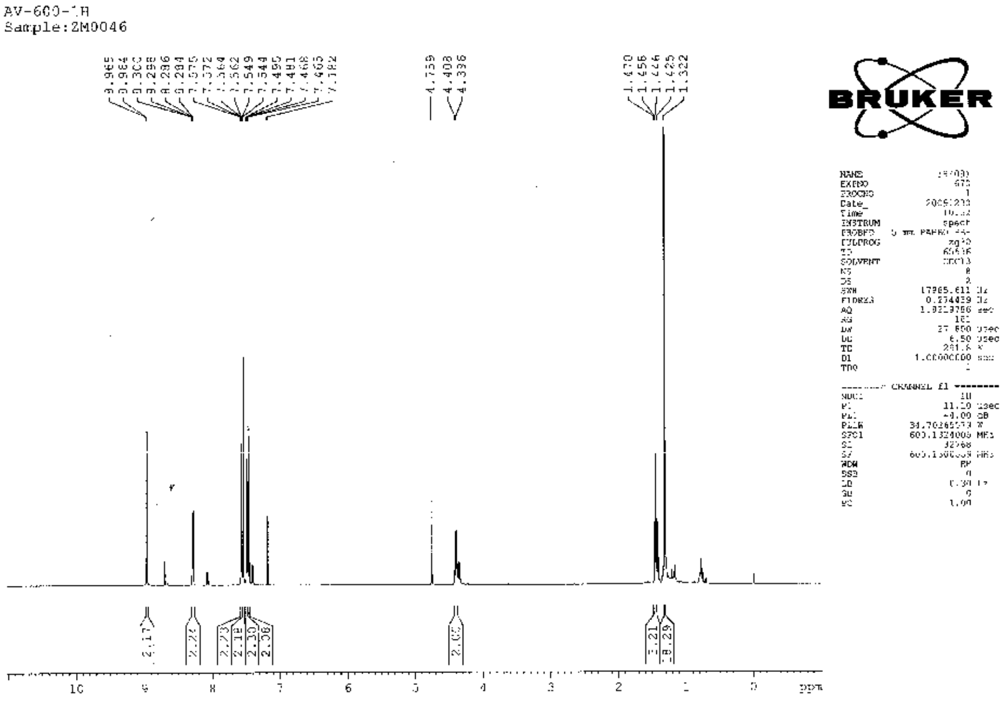

Figure S40: ^{1}H NMR spectrum of **5j**.

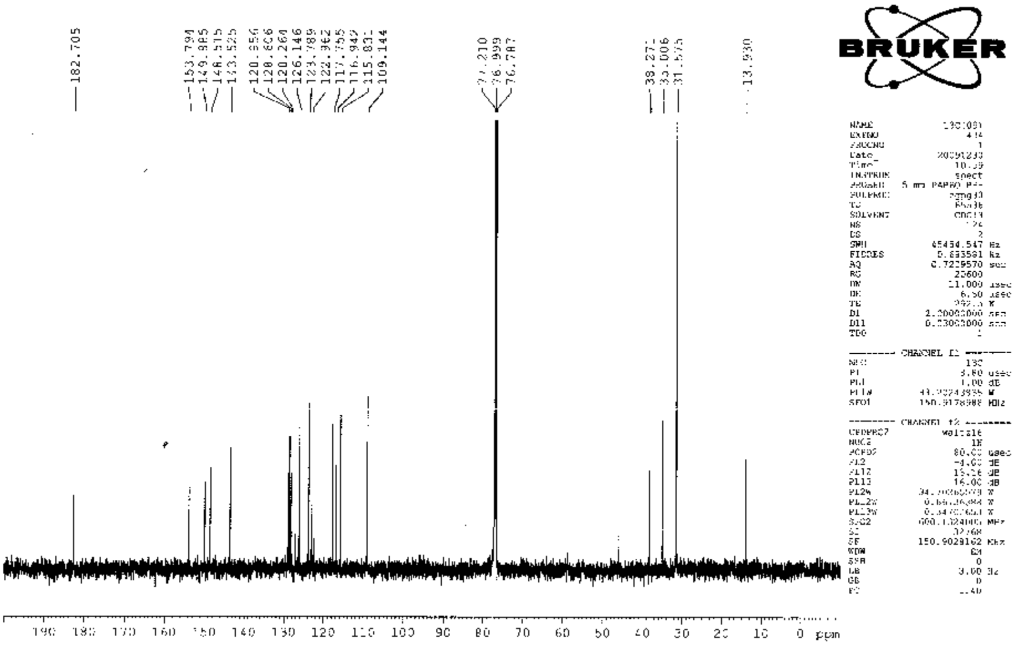

Figure S41: ^{13}C NMR spectrum of **5j**.

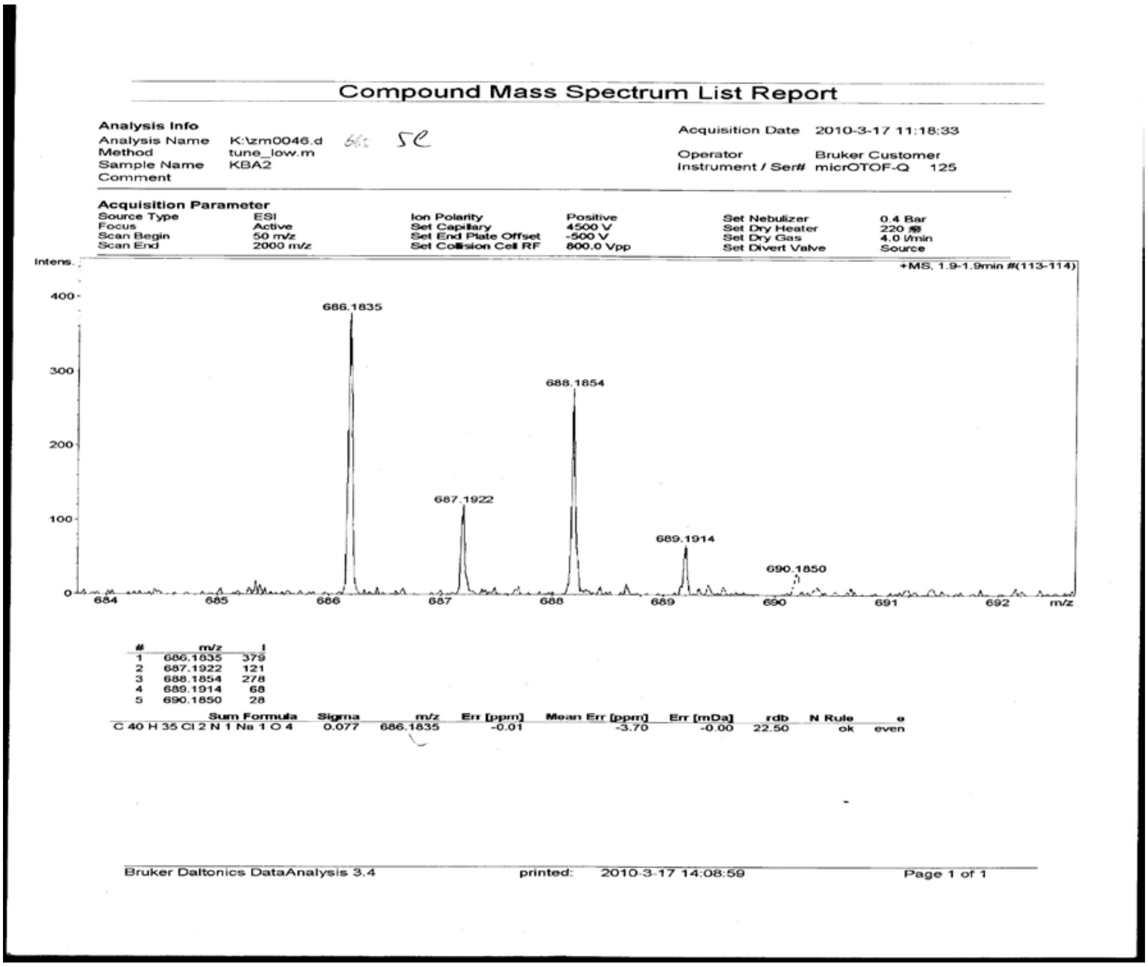

Figure S42: HRMS spectrum of **5j**.

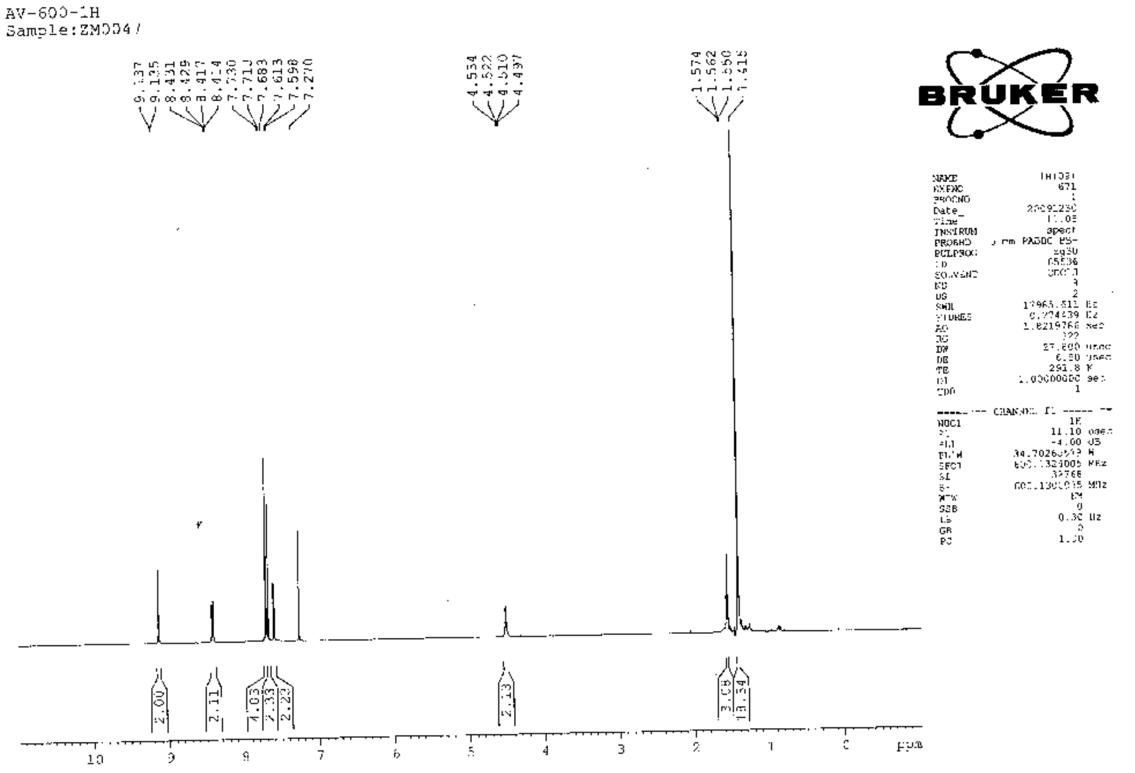

Figure S43: ^{1}H NMR spectrum of **5k**.

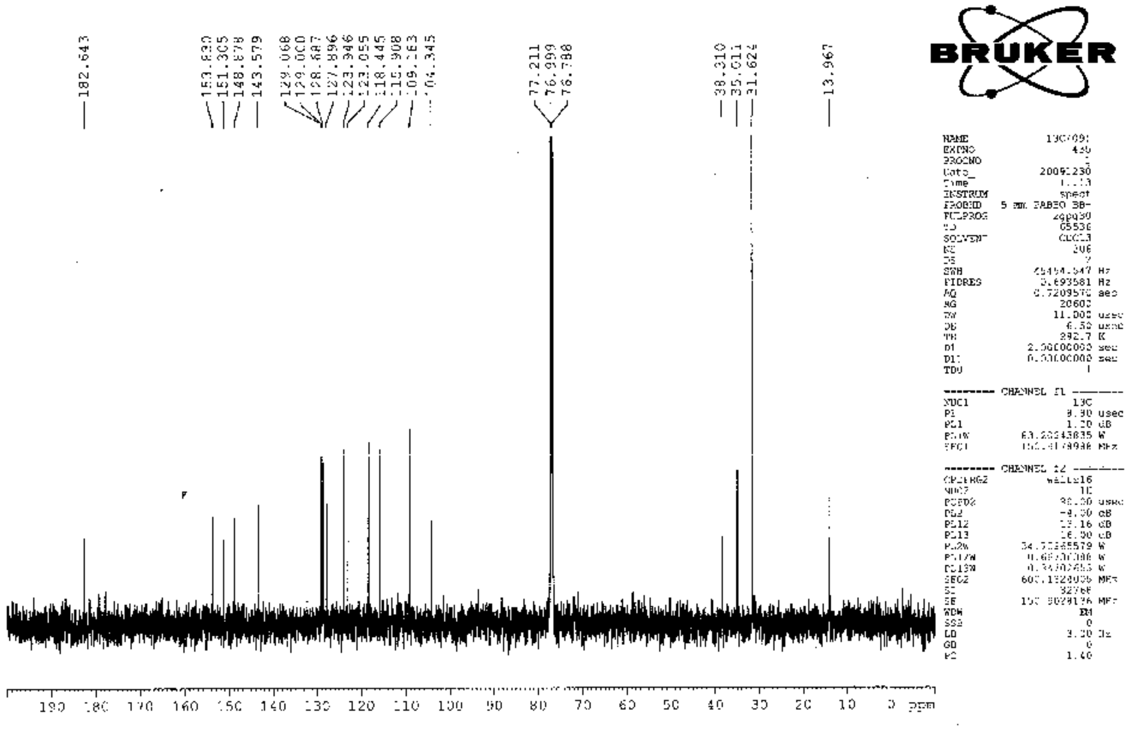

Figure S44: ^{13}C NMR spectrum of **5k**.

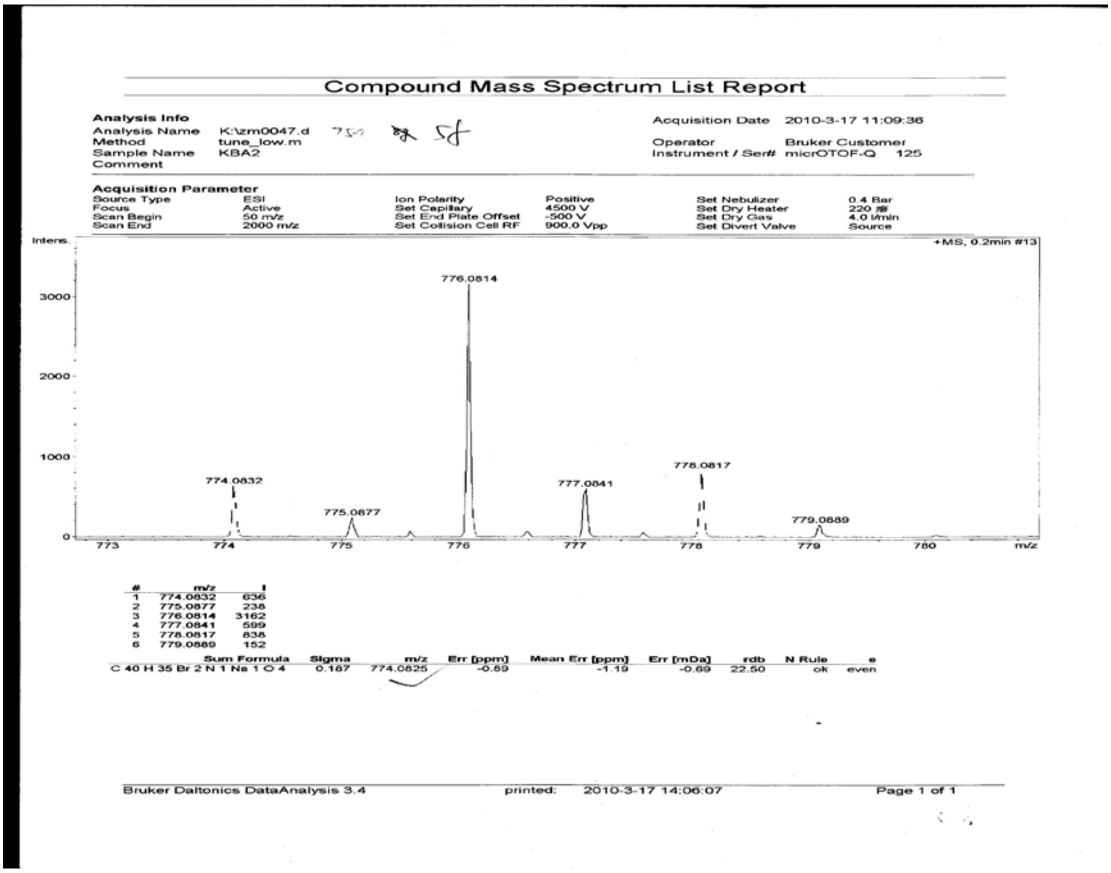

Figure S45: HRMS spectrum of **5k**.

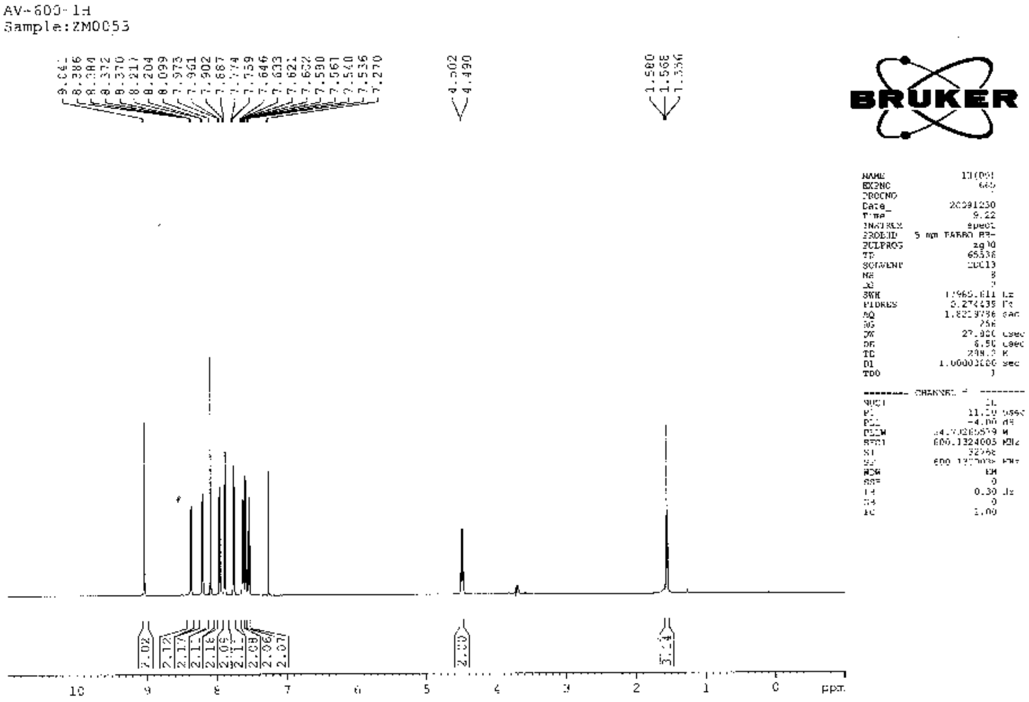

Figure S46: ^{1}H NMR spectrum of **5l.**

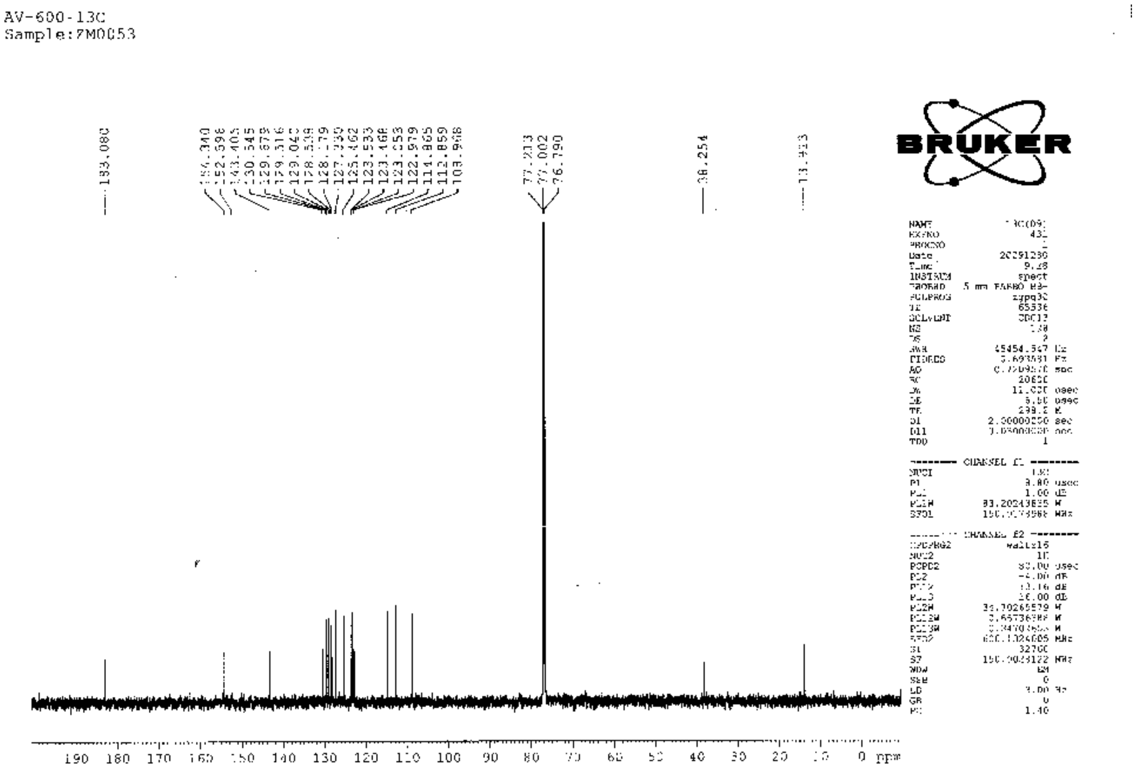

Figure S47: ^{13}C NMR spectrum of **5l**.
